# Atomistic structure of the SARS-CoV-2 pseudoknot in solution from SAXS-driven molecular dynamics

**DOI:** 10.1093/nar/gkad809

**Published:** 2023-10-11

**Authors:** Weiwei He, Josue San Emeterio, Michael T Woodside, Serdal Kirmizialtin, Lois Pollack

**Affiliations:** Chemistry Program, Science Division, New York University, Abu Dhabi, United Arab Emirates; Department of Chemistry, New York University, USA; School of Applied and Engineering Physics, Cornell University, USA; Department of Physics, Li Ka Shing Institute of Virology, and Centre for Prions and Protein Folding Diseases, University of Alberta, Canada; Chemistry Program, Science Division, New York University, Abu Dhabi, United Arab Emirates; Department of Chemistry, New York University, USA; School of Applied and Engineering Physics, Cornell University, USA

## Abstract

SARS-CoV-2 depends on −1 programmed ribosomal frameshifting (−1 PRF) to express proteins essential for its replication. The RNA pseudoknot stimulating −1 PRF is thus an attractive drug target. However, the structural models of this pseudoknot obtained from cryo-EM and crystallography differ in some important features, leaving the pseudoknot structure unclear. We measured the solution structure of the pseudoknot using small-angle X-ray scattering (SAXS). The measured profile did not agree with profiles computed from the previously solved structures. Beginning with each of these solved structures, we used the SAXS data to direct all atom molecular dynamics (MD) simulations to improve the agreement in profiles. In all cases, this refinement resulted in a bent conformation that more closely resembled the cryo-EM structures than the crystal structure. Applying the same approach to a point mutant abolishing −1 PRF revealed a notably more bent structure with reoriented helices. This work clarifies the dynamic structures of the SARS-CoV-2 pseudoknot in solution.

## Graphical Abstract

**Graphical Abstract:**
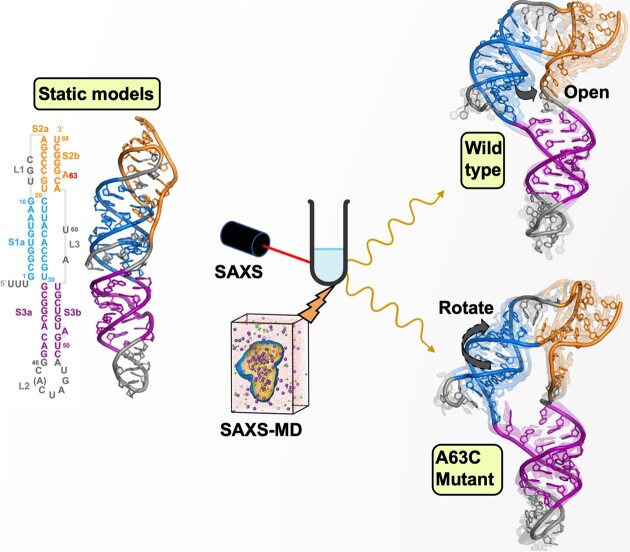

## Introduction

The virus causing COVID-19, SARS-CoV-2, makes use of the translational recoding mechanism known as −1 programmed ribosomal frameshifting (−1 PRF) to express essential proteins (1). In −1 PRF, the ribosome is directed to shift into the −1 reading frame at a specific location in the viral mRNA by the presence of a frameshift signal consisting of a 7-nucleotide (nt) slippery sequence positioned ∼5–7 nt upstream of a stimulatory structure (2). In the case of coronaviruses like SARS-CoV-2, the frameshift is stimulated by a pseudoknot (3,4); it is required for the ribosome to access the adjacent reading frame, which codes for non-structural proteins 12–16, including the viral polymerase necessary for replication (1,5). Given that disruption of normal −1 PRF levels attenuates coronavirus (CoV) propagation significantly (6–10) and that the frameshift-stimulatory pseudoknot is among the most conserved regions in CoV genomes (4,11), the pseudoknot is a promising target for novel anti-viral drugs to treat COVID-19. (1) Indeed, a number of studies have reported small-molecule ligands that are effective at modulating −1 PRF in SARS-CoV-2 (3,9,10,12–14) as well as other CoVs (9,10,13).

**Figure 1.**
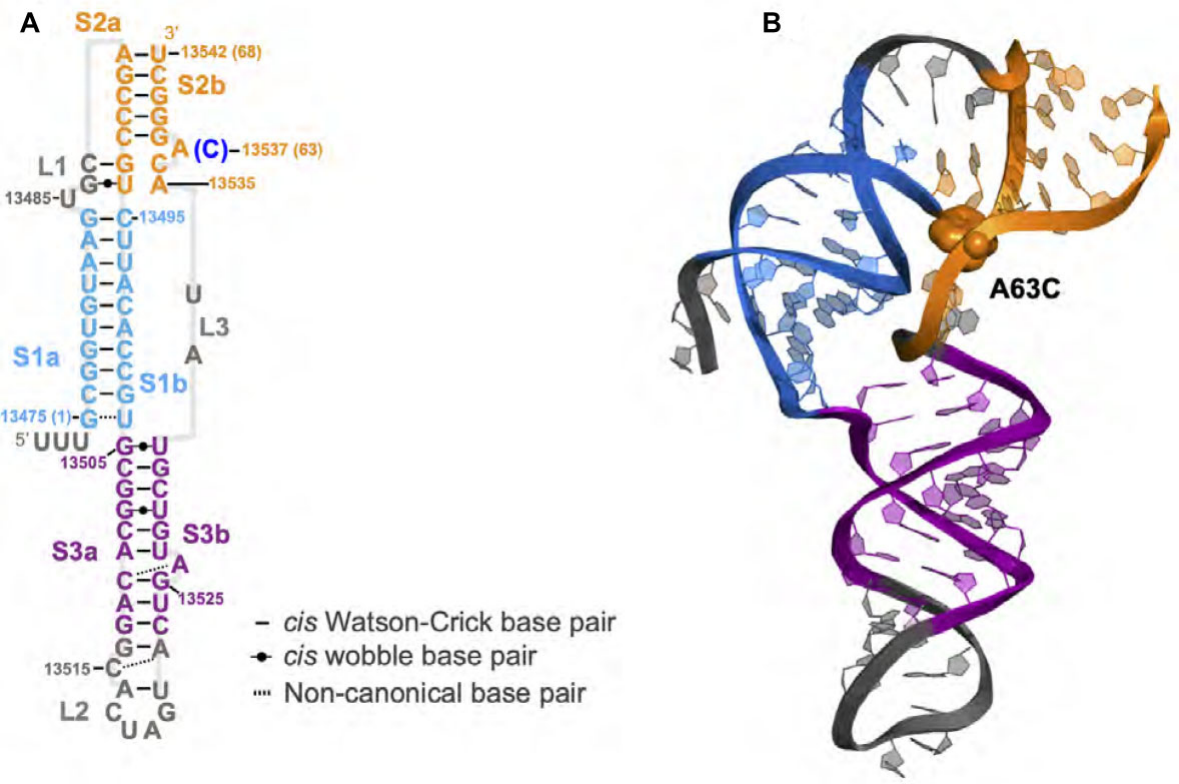
The SARS-CoV-2 frameshifting pseudoknot. Secondary structure from Zhang *et al.* (17) is shown in panel (**A**). The corresponding tertiary structure is shown in panel (**B**). Stem 1 (S1, created by base pairing of S1a and S1b) is shown in blue, stem 2 (S2, with strands S2a and S2b) is shown in orange, and stem 3 (S3, with strands S3a and S3b) is shown in purple. All loops, designated L1–3, are shown in grey. The position of the A63C mutation is highlighted in the secondary and tertiary structures. Numbering in panel (A) shows position in SARS-CoV-2 genome; numbers in brackets denote position within pseudoknot.

Because the RNA frameshifting element is small and well structured, drug discovery and optimization can be accelerated by knowledge of the pseudoknot’s solution structure. Whereas most frameshift-stimulatory pseudoknots have a simple 2-stem H-type architecture (15,16), the SARS-CoV-2 pseudoknot has a more complex 3-stem architecture (Figure 1A) (3,17). Recent experimental (17–22) and computational (23–26) work investigating the structure and dynamics of this pseudoknot has revealed that it can assume multiple conformations and fold topologies. The dominant conformer involves an unusual topology wherein the 5′ end of the RNA is threaded through a ring formed by the 3-stem junction and closed by the pseudoknot interactions in stem 2 (S2) (Figure 1B). This prior work has provided significant insight into the structural properties of the pseudoknot and how they may relate to frameshifting mechanisms. However, the experimentally obtained high-resolution structures differ from one another, likely as a result of different experimental techiques and measurement conditions. The reported structures were obtained by cryo-EM studies of the isolated pseudoknot at high Mg^2 +^ (17) or the pseudoknot on a stalled ribosome (19). Crystallographic structures were obtained of the isolated pseudoknot (20) or the pseudoknot complexed with a chaperone (21). None of these structures characterized the pseudoknot in solution conditions, similar to what would be expected physiologically. As a result, a consensus on the structure that could serve as a drug target remains elusive.

Small-angle X-ray scattering (SAXS) offers a powerful approach for exploring solution structures of macromolecules, enabling comparison with structures obtained from rigid or frozen samples by methods like cryo-EM and crystallography (17,19,20). In SAXS, the intensity of X-rays scattered from molecules suspended in solution is measured as a function of angle and used to probe the global size and shape of the molecule (27,28). SAXS profiles can distinguish bent from linear conformations, for example SAXS measurements identified the salt-dependent bent states of model RNA helix–junction–helix constructs (29). Although SAXS alone does not provide high resolution, it can be integrated with computational methods such as in SAXS-driven molecular dynamics (MD) simulations (30,31) to generate models of the structure in solution.

Here, we present SAXS measurements of the SARS-CoV-2 frameshift-stimulatory pseudoknot, which we combined with SAXS-driven MD to build atomic models of the pseudoknot structure in solution. We found that none of the scattering profiles computed from atomic coordinates for the previously solved structures was consistent with the measured SAXS profile: all were too linear in conformation, with the disagreement largest for the crystal structure. However, SAXS-driven MD refinement of the static structures led to new models whose computed scattering profiles agreed reasonably well with the measured profile. Repeating the analysis for a point mutant that is frameshift-incompetent, A63C (6), we found that the mutation produced a significant change in the SAXS profile, reflecting an even stronger bend in the structure at the 3-stem junction. These results clarify that linear conformations of the SARS-CoV-2 pseudoknot are disfavored in solution, with the dominant conformers featuring a notable bend at the ring through which the 5′ end of the transcript is threaded. Our study provides atomistic models of the solution structure consistent with the experiment.

## Materials and methods

### Pseudoknot sample preparation

Plasmids containing the appropriate sequences were produced according to Kelly *et al.* (3). The slippery sequence is unfolded inside the ribosome when frameshifting occurs and thus does not contribute to the structure that stimulates frameshifting, hence was excluded from the RNA sequence (Supplementary Table S1). Six nucleotides comprising the spacer region at the 5′ end were included, however, to allow for threading of the 5′ end (22,23). The template sequence was then PCR amplified to include a T7 polymerase initiator and the appropriate termination. This template was used for *in vitro* transcription using Hiscribe (NEB) following the manufacturer’s directions. The RNA product was ethanol precipitated, then re-suspended in a low ionic strength buffer (50 mM MOPS 130 mM KCl) and annealed by heating to 95°C for 5 min before placing it on ice. The sample was then concentrated and used for size-exclusion chromatography SAXS (SEC-SAXS).

### Solution X-ray scattering and evolving factor analysis

SAXS experiments were performed at the life sciences X-ray scattering (LIX) beamline at Brookhaven National Laboratory using their standard SAXS configuration (32). As described in that publication, the scattering intensity, *I* is measured at different angles or *q* = 4πsinθ/λ, where 2θ is the scattering angle and λ is the X-ray wavelength. Data were visualized, processed, and analyzed using RAW (33). To isolate the scattering from an individual molecular fraction, in this case, monomers, SEC-SAXS was performed using a superdex 200 5/150 column (Cytvia) pre-equilibrated in a buffer of 50 mM MOPS 130 mM KCl pH 7.5 To compensate for the presence of overlapping species within the measured elution volumes, further isolation of fractions can be achieved computationally, using evolving factor analysis performed through RAW following Refs (33–35). In this way, the signal of any specific fraction, in this case, the monomeric species, can be uniquely extracted. We note that SEC-SAXS does not have sufficient resolution to separate different conformations within the monomer population, hence the signal from the monomer fraction represents an average over all monomeric conformations.

### Structural modeling and simulation details

We used molecular dynamics (MD) simulations to generate conformational ensembles of the pseudoknot structures as detailed in SI Methods (section on General MD simulations set up). We constructed pools starting from cryo-EM models of the wild-type SARS-CoV-2 pseudoknot solved in isolation (PDB ID: 6XRZ (17)) or in the presence of the ribosome (PDB ID: 7O7Z (19)), as well as from a crystallographic structure of the pseudoknot in isolation (PDB: 7LYJ (20)). The initial model obtained from the crystallographic structure (PDB ID: 7LYJ (20)) was adjusted with PyMOL (36) to more closely match the sequence used in SAXS, and during this process, the 5′ end was extended by 3 nt, to allow for threading of the 5′ end (see SI). As the structure of the A63C mutant has not been solved experimentally, we mutated the A63 to C computationally in both cryo-EM models and performed MD simulations for structural refinement (Supplementary Tables S2 and S3). We computed the scattering profile expected from the simulated structural ensembles following (37–39), for comparison with the measured signal, with an explicit treatment of water molecules, condensed counter ions, and the RNA conformational ensemble (Supplementary Figure S1). Full details of the approach are provided in SI Methods (section on Computing SAXS from MD trajectory).

To refine the simulated structures through comparison to the measured scattering profiles, we biased the conformations generated by MD to meet the experimental signal by adding a penalty term to the energy function used in the simulations *E*_*hybrid*_ = *E*_*FF*_ + *E*_*SAXS*_, where *E*_*FF*_ is the energy from the MD force field and *E*_*SAXS*_(*t*) is a time dependent penalty term arising from differences between the computed and measured profiles, *I*_*comp*_(*q*, *t*) and *I*_*exp*_(*q*) amplitudes given as:


(1) \begin{eqnarray*} E_{SAXS}(t) = \alpha (t)k_c \frac{k_BT}{n_q}\sum _{i=1}^{n_q} \frac{\left\lbrace I_{comp}(q_i,t) - I_{exp}(q_i) \right\rbrace ^2}{\sigma ^2(q_i)}. \end{eqnarray*}

Here *n*_*q*_ is the number of intensity points spanning the specific range of scattering vectors *q*. In this study, the constructs were refined against SEC-SAXS data up to q = 0.315 Å^−1^. *k*_*c*_ is a weighting term for *E*_*SAXS*_ compared to *E*_*FF*_, and α(*t*) is a time-dependent function that allows the penalty function to be turned on gradually during the simulation. The uncertainties in the measurement and in the back calculation of the scattering profile were added in quadrature: $\sigma ^2(q_i)=\sigma _{exp}^2(q_i)+\sigma _{comp}^2(q_i)+\sigma _{buffer}^2(q_i)$, where $\sigma _{exp}^2(q_i)$, $\sigma _{comp}^2(q_i)$ and $\sigma _{buffer}^2(q_i)$ are respectively the experimental error, statistical error from computed curves, and systematic errors due to the uncertainty of buffer density (30,31,38,40).

### Data analysis

To quantify the goodness of the refinement, we used the χ^2^ metric,


(2) \begin{eqnarray*} \chi ^2 = \frac{1}{n_q-1}\sum _{i=1}^{n_q} \left\lbrace \frac{ I_{exp}(q_i) - I_{comp}(q_i) }{\sigma _{exp}(q_i)} \right\rbrace ^2 \end{eqnarray*}

where *I*_*exp*_(*q*_*i*_), *I*_*comp*_(*q*_*i*_) are scattering intensities from experiment and simulation respectively, σ_*exp*_(*q*_*i*_) presents the experimental error.

From the SAXS-driven MD simulations, we went over all the frames and selected the snapshots below a threshold value, χ^2^ < 4.0 in our case. These conformations dominate the ensemble and exhibit the highest contribution to the average SAXS profile (see Supplementary Figure S2). As a result, they serve as the conformational ensemble to perform further analysis. The lowest χ^2^ value conformation represents the SAXS profile in each simulation setup. As MD simulations conserve energy, the lowest χ^2^ value conformation serves as the lowest energy state of the extended Hamiltonian. The result is a structural pool consisting of over 20000 conformations for each system.

To compare the conformational ensembles and distinguish the structural differences between them, we employed contact map analysis. Briefly, for a given RNA conformation, at time *t*, the contacts are considered between residue *i* and *j* when the shortest distance between residues *s*_*ij*_(*t*) < *r*_*c*_, where *r*_*c*_ is the critical distance for contact formation and is set to 8 Å in this study. Then, the time average of contact formation probability *C*(*i*, *j*) was calculated and used to make the contact maps; more information on the method is given in SI.

To determine the critical changes in pseudoknot structures during SAXS-driven MD refinement, we conducted a principal component analysis (PCA) using the Gromacs implementation. Supplementary Figure S5 depicts the structural changes in the pseudoknot, projected onto the eigenvectors of the two highest eigenvalues (PCA vec 1 and PCA vec 2) during the refinement process. Further information on the analysis is available in the supplementary information section.

We classify the RNA structures sampled by SAXS-driven MD using the spectral clustering algorithm (41). For that, each pair of conformations are optimally aligned and their RMSD were computed using MDTraj package (42), resulting in an N×N square matrix of pairwise RMSD values. Subsequently, structures with an RMSD <10  Å are considered related, and based on that the RMSD matrix is converted to a binary (0/1) adjacency matrix, encoding the connectivity information between pairs of RNA structures. The RMSD-derived adjacency matrix is then passed to the spectral clustering algorithm which maps RNA structures to graph space, and K-means is used to assign labels in the embedding space, leading to subgroups of RNA structures. The clustering analysis is performed utilizing the Scikit-learn Spectral clustering algorithm (41,43).

## Results

**Figure 2.**
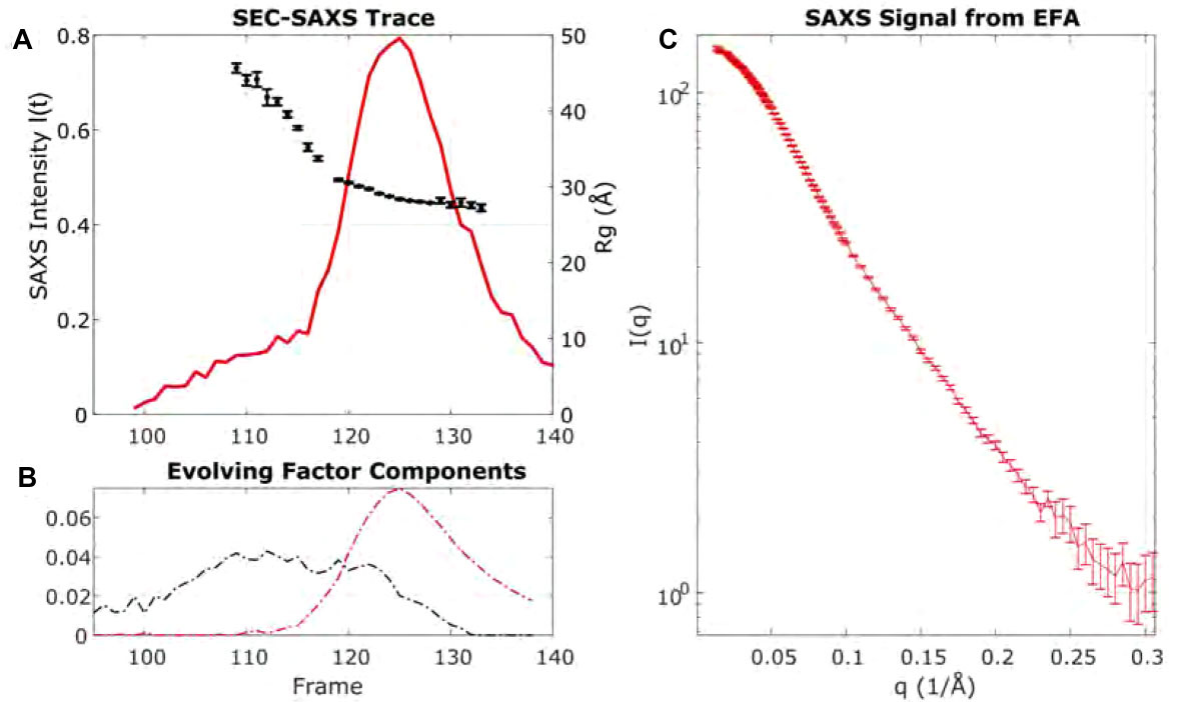
Size exclusion chromatography SAXS data of wild-type construct. (**A**) The size exclusion trace from the integrated SAXS intensity is shown (left axis, red curve). The intensity changes as a function of time as molecules elute from the column and flow into the sample cell for examination by X-rays. At early elution times, a low shoulder in the red curve signals the presence of molecules in the sample with large and decreasing Rg (black symbols, right axis). A single large elution peak appears just before frame 120, containing molecules whose radius of gyration (Rg black symbols, right axis) is comparable to that previously reported for the monomeric pseudoknot (3). (**B**) Evolving factor analysis indicates that the sample eluting in the large peak still contains a mixture of species. Further analysis allows for extracting the monomer scattering profile (red component in this panel). The extracted SAXS profile of the monomer is shown to the right in panel (**C**). SEC-SAXS/EFA results for the mutant construct can be found in Supplementary Figure S3.

Solutions containing the SARS-CoV-2 pseudoknot at concentrations required for SAXS contained a mixture of differently sized structural species, which was expected because the pseudoknot is known to dimerize (44). Dimerization was reduced but not eliminated by measuring in the absence of Mg^2 +^. Therefore, to isolate the scattering signature of the monomer fraction for direct comparison to simulation, we applied size exclusion chromatography (SEC-SAXS) to separate the different species (Figure 2A, red). Because the peaks are not completely separated (the elution fractions are not discrete), we then applied evolving factor analysis (EFA) (34,35) to extract the monomer profile from those reflecting higher-order structures (Figure 2B, red). The scattering profile of the monomer fraction is displayed in Figure 2C as *log*(*I*) vs. *q*.

**Figure 3.**
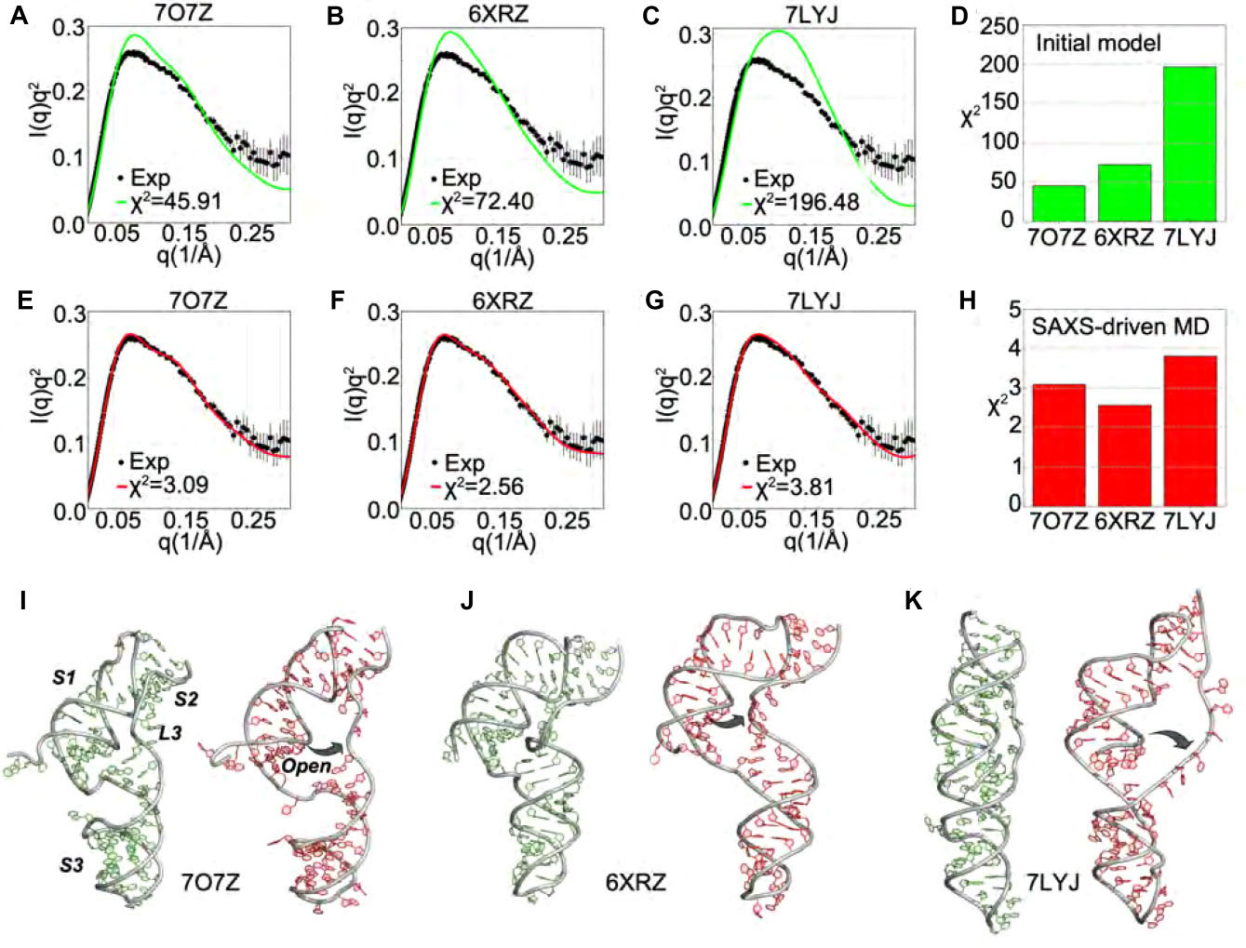
Comparison of wild-type pseudoknot structure models before and after SAXS-driven MD simulations. The fits to the scattering data from different structures are shown in panels (**A**)-(**C**). The computed χ2 for the initial structures is shown in panel (**D**).The fits to the scattering from the SAXS-driven MD are shown in panels (**E**)–(**G**). The computed χ2 from the SAXS-driven MD structures is shown in panel (**H**). Note that panels (**D**) and (**H**) have different axes (the χ2 from the SAXS-driven MD is much lower). The residuals of the fitting are summarized in Supplementary Figure S6. A comparison of the initial (green) with SAXS-Driven MD (red) is shown in panels (**I**)–(**K**): (**I**) PDB:7O7Z (19), (**J**) PDB:6XRZ (17) and (**K**) PDB:7LYJ (20). The black arrows indicate loop 3 opening in all cases, which is also the most predominant change captured by the PCA analysis (see PCA vec 1, Supplementary Figure S5).

To determine if the monomer scattering profile was consistent with published structures for the SARS-CoV-2 pseudoknot, we computed the expected scattering profile from three different structural models: a cryo-EM structure of the pseudoknot complexed with a ribosome arrested in translation at the slippery site (PDB ID 7O7Z) (19), a cryo-EM structure of the pseudoknot in isolation at high Mg^2 +^ (PDB ID 6XRZ) (17), and a crystal structure of the pseudoknot in isolation (PDB ID 7LYJ) (20). The computed profiles for each of these models are displayed as Kratky plots of *Iq*^2^ versus *q* to aid in the visualization of subtle differences in the profiles at larger *q* where the spatial resolution is highest (Figure 3A–C, green). Significant deviations from the measured profiles are apparent (Figure 3A–C, black): the former is consistently higher than the measured profile for q values in the range *q* ∼ 0.07–0.15 Å^−1^, and consistently lower than the measured profile above *q* ∼ 0.18 Å^−1^. The cryo-EM model on the ribosome yielded the most similar profile to the measured one, producing the lowest χ^2^ (Figure 3D). The cryo-EM model of the isolated pseudoknot yielded a similar result, whereas the crystal structure displayed the largest deviations.

Given that none of the profiles computed from published structures agreed well with the measurement, we used these structures as starting points for SAXS-driven MD simulations. SAXS-driven MD searches the conformational space near the starting structures to find conformations whose computed scattering profiles better agree with the SAXS measurements. This method reveals how the initial structures need to change to improve agreement with measurement. The dramatic improvements in the agreement are illustrated in (Figure 3E–G) and reflected by sharply lower χ^2^ values (Figure 3H). All three profiles generated from the modified structures captured the essential features observed experimentally, as confirmed by analysis of the residuals from comparing models and experiment (Supplementary Figure S6). Nevertheless, the models initiated from cryo-EM structures achieved slightly lower χ^2^ values than the model initiated from the crystal structure, suggesting that these models more closely resemble the solution structure.

Figure 3I–K (red) shows 3D structures with the lowest χ^2^ obtained from SAXS-driven MD. The structure that is pictured represents the lowest energy structure, which is likely the most abundant conformation in the ensemble. Also shown are the starting structures used in the simulations (Figure 3I–K, green). Differences between the two reveal the changes needed to gain good agreement with the observed SAXS profile. For the two cryo-EM structures (7O7Z and 6XRZ, respectively Figure 3I and J), only small structural changes were required to drastically improve the agreement between computed and measured profiles. Most notably, in these models L3 moved slightly to open up the ring at the 3-stem junction through which the 5′ end was threaded. Additionally, S1 was modified very slightly (Supplementary Figure S5). The detailed mechanism of these conformational transitions are displayed in Supplementary Figure S4 using PCA analysis. More substantial changes were required when starting from the crystal structure determined coordinates (7LYJ, Figure 3K). Here, the almost linear arrangement of stems 1–3 with L3 tightly packed along the side was disrupted by bending stem 3 with respect to stem 1, distorting the stacking between S1 and S2. The ring at the 3-stem junction was opened by moving L3 away from S1.

**Figure 4.**
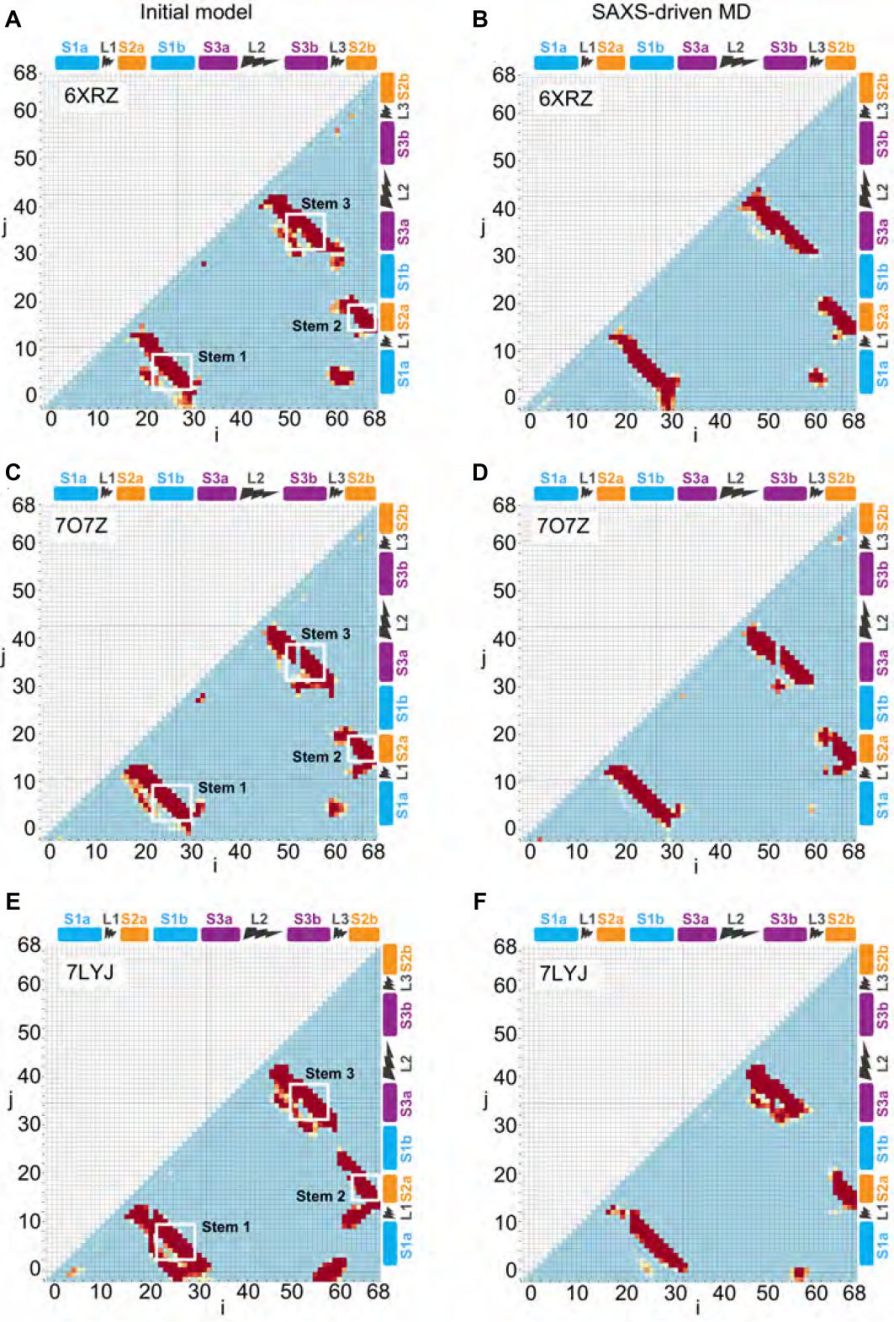
Contact maps for the wild-type pseudoknot structures. Model 6XRZ is shown in (**A**), (**B**), model 7O7Z is shown in (**C**), (**D**), and model 7LYJ is shown in (**E**) and (**F**). The contact maps on the left are built from a conformational ensemble sampled by unrestrained MD from the initial cryoEM structure. In contrast, the right ones are sampled by SAXS-driven MD. Colors represent the different probability of contacts (Eqs. (3) and (4) of contact map analysis in SI), the red region has the highest probability, and the blue area indicates the lowest probability. The difference map between initial models and SAXS-driven MD pool can be found in Supplementary Figure S9.

To characterize the structural differences between the initial and final structures in the SAXS-driven MD process, we computed contact maps, which allow the visualization of tertiary contacts and long-range distance correlations within the structures. For the SAXS-driven MD results, the structural pools used to generate contact maps were taken from the refinement trajectories; for the cryo-EM/crystallographic structures, we used trajectories from standard MD simulations starting with the reported structures with no refinement. In the contact maps for the initial structures (Figure 4, left column), the base-paired regions forming stems as defined in Figure 1 are seen as the dark diagonal lines with slope −1 bounded by white frames. These diagonals extend beyond the nominal boundaries of the stems (S1-3), reflecting limited flexibility of the nucleotides in L1 (at the top of S1) and L2 (at the bottom of S3) such that they remain in close proximity. Beyond these boxed regions, proximity of residues (but not necessarily base-pairing) is detected between S1a and S2a (lower left part of contact map) and between S1a and L3 (bottom right). This proximity reflects a pinching in of L3 in the PDB structures, which in turn affects the orientation of S2. Another common feature is an additional diagonal with slope −1 just below S3 (which is closed by the hairpin loop L2), indicating proximity to regions that are slightly separated in sequence but not base-paired into the stem. The above-described features are present in all of the starting structures.

The contact maps also reveal notable differences between the PDB deposited structures. These are most clearly visualized by computing difference maps between pairs of structures (Supplementary Figure S7). Comparing first the two cryo-EM structures, the difference map shows that a few of the contacts moved, implying modest structural rearrangements. For example, contacts between S1a and S1b in 6XRZ moved to S1a and S2a in 7O7Z; similarly, contacts between S3a and S3b moved to S3a and L2. Additional contacts were also formed between S1b and L3. In contrast, more substantial changes are evident when comparing 6XRZ to 7LYJ. Among the many differences, new contacts formed in the latter between S1 and S2, between L1 and L3 and between S1a and S3b.

Turning next to the contact maps for the SAXS-driven MD ensembles, we found much less variation among the contacts formed in the final ensembles (Figure 4, right column). Although the contact maps for the refined structures are not identical, they share very similar features. This provides confidence that the SAXS-driven MD refinements result in similar shapes and tertiary contacts, despite the significant differences in the starting structures. Using difference maps to compare the three models (Supplementary Figure S8), we found that most of the deviations between the refined structures occurred in the loop or terminal regions of the pseudoknot, presumably reflecting their greater conformational flexibility.

**Figure 5.**
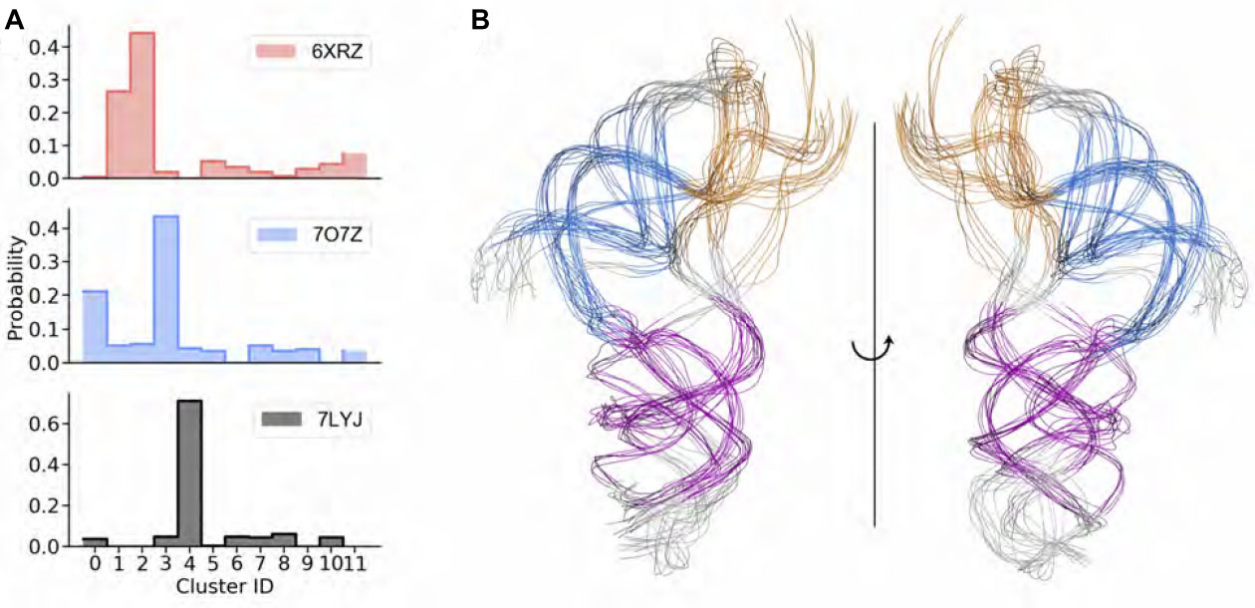
Clustering analysis of the pseudoknot conformational ensemble. (**A**) Cluster distributions within each ensemble suggest dynamic flexibility and shared space. (**B**) The overlay of representative RNA conformations representing cluster centers.

To ascertain that the differences between the SAXS-driven MD results starting from different structures were not due to the simulations becoming trapped in local minima, we analyzed the set of conformational clusters visited in each trajectory. We first merged the three pools of SAXS-driven simulations and performed spectral clustering (41) on the combined ensemble to identify the set of conformational clusters visited by the pseudoknot in all simulations. We then clustered each simulation individually, to determine the distribution of conformational clusters visited in each simulation (Figure 5A). Each simulation starting from a different initial structure had different dominant clusters, but also showed overlap in the clusters visited, indicating dynamic fluctuations into regions visited by the other models. The representative structures of the conformational clusters are illustrated in Figure 5B.

**Figure 6.**
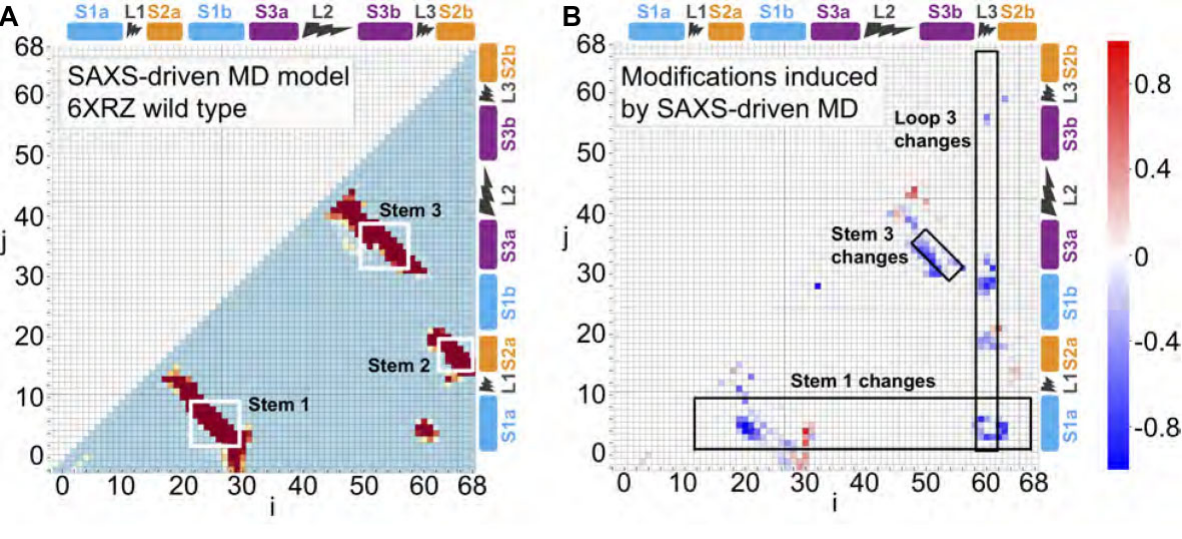
Contact and difference maps for the wild-type pseudoknot structure. (**A**) The contact map of the SAXS-driven MD ensemble for the wild-type pseudoknot structure. Colors represent the probability of contacts. The two residues are defined as in contact when the shortest distance between their atoms is less than 8 Å. (**B**) The differences in contact probabilities before and after SAXS-driven MD ensembles. The initial ensembles were constructed by brute force MD starting from PDB id 6XRZ. Rectangular frames highlight the major modifications after SAXS-driven MD. The probability differences are colored as a heat map; blue marks the loss, and red marks the formation of new contacts.

The most interesting results from the contact map analysis, however, arose from comparing the initial and final structures, indicating the changes required to bring the profiles computed from refined PDB structures into better agreement with the solution measurement. Focusing on the model that nominally fit best, the one derived from simulations starting with the cryo-EM structure 6XRZ (Figure 6A), we found that the difference map (Figure 6B) was dominated by a loss of contacts in multiple regions. The most significant changes involved S1, L3, and S3. The majority of changes to the S1 region involve significant loss of contacts (blue pixels) with the exception of the region of S1b (residues 28–30) with the 5′ end (residues 2–5, S1a), closing the stem S1 (Supplementary Figure S5B). These changes were associated with the expansion of the ring around the 3-helix junction as L3 moved away from the other helices. The reduction in contacts within L3 was complementary with this picture, with the proximity decreasing between L3 and regions of the 3-helix junction closest to it: the lower parts of S1a and S1b, the lower part of S2a, and to a lesser extent, the upper parts of S3a and S3b. This result is in accord with the principle component analysis (Supplementary Figure S5), highlighting the extent of change in loop 3 in the solution structure. The third region showing notable loss of contacts was S3, which lost contacts to residues outside of S3, including those in the terminal loop, reflecting a reorientation of L2 closing the hairpin and supporting the enlargement of the ring at the center of the structure. In contrast, proximity increases were seen within L2, and most notably between residues at the bottom of stem 1, indicating closer coupling and perhaps the formation of new contact pairs. As noted above, these changes in S1 reflected tighter proximity of the two strands in S1 as it pulled away from S3 and L3 with the enlargement of the ring. Finally, we compared the flexibility of the pseudoknot in the intial and final structures by calculating the *B*-factor (Supplementary Figure S11), finding that they were qualitatively similar, showing relatively higher flexibility in L2, L3 and the 5′ end of S2 compared to the other loops and stems.

Comparable effects were seen in the difference maps for the final and initial structures for the other two models, derived from 7O7Z and 7LYJ (Supplementary Figure S9): the difference maps were dominated by loss of contacts in similar regions. However, the loss of contacts was much more extensive and severe for the crystallography model (Supplementary Figure S9C); the latter also featured some gains of contacts within S3, between S3a and L3, and between S1b and L1. *B*-factor analysis was not meaningful for 7O7Z owing to the relatively low resolution of the structure, but for the crystal structure (7LYJ) again showed elevated flexibility for L3 and the 5′ end of S2; the results differed from above in showing low fluctuations for L2 and elevated flexibility in L1. Note that all solution refined structures presented here had substantial variations of the flexible parts of the molecule relative to the static structures, particularly in the region of L3 (responsible for setting the bending angle of the fold), consistent with the structures shown in Figure 3. Intriguingly, this region has been identified as potentially key conformational switch in a recent computational study (26).

**Figure 7.**
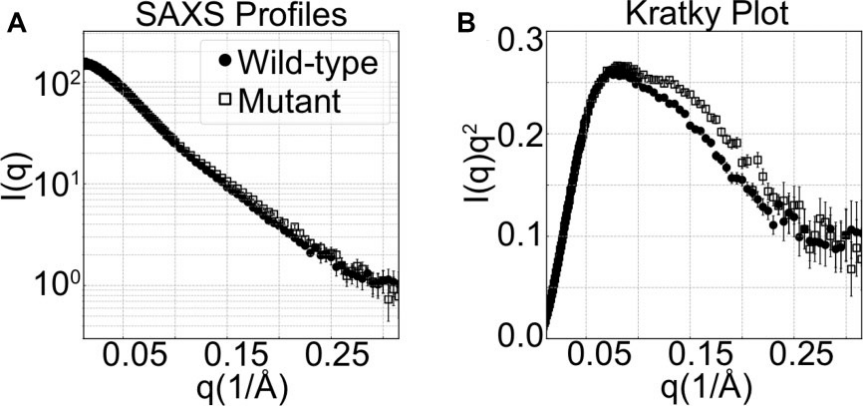
Comparison of SEC-SAXS data for the wild-type and mutant pseudoknot. (**A**) The raw SAXS profiles, in *I* versus *q* representation, (**B**) as Kratky plots.

We next turn to the structural impact of a single-point mutation, A63C. This mutation is of particular interest as it was found to abolish frameshifting in the SARS-CoV pseudoknot (6). In contrast to the wild-type SARS-CoV-2 pseudoknot, no structural information is available for this mutant. Although the SAXS profiles for wild type and mutant constructs are effectively identical at low angles, *q* < 0.07 Å^−1^, the latter shows a distinct and systematic increase in intensity above *q* ∼ 0.1 Å^−1^ (Figure 7A). This deviation is seen most clearly in the Kratky plot, which reveals a weak, secondary peak in the range *q* ∼ 0.1−0.15 Å^−1^ (Figure 7 B). Based on previous studies of short duplexes joined by flexible linkers (29), these changes to the scattering profile suggest an enhanced bending angle between helices in the structure. The mutation thus appears to increase the bend between S1 and S3 relative to the wild-type structure.

**Figure 8.**
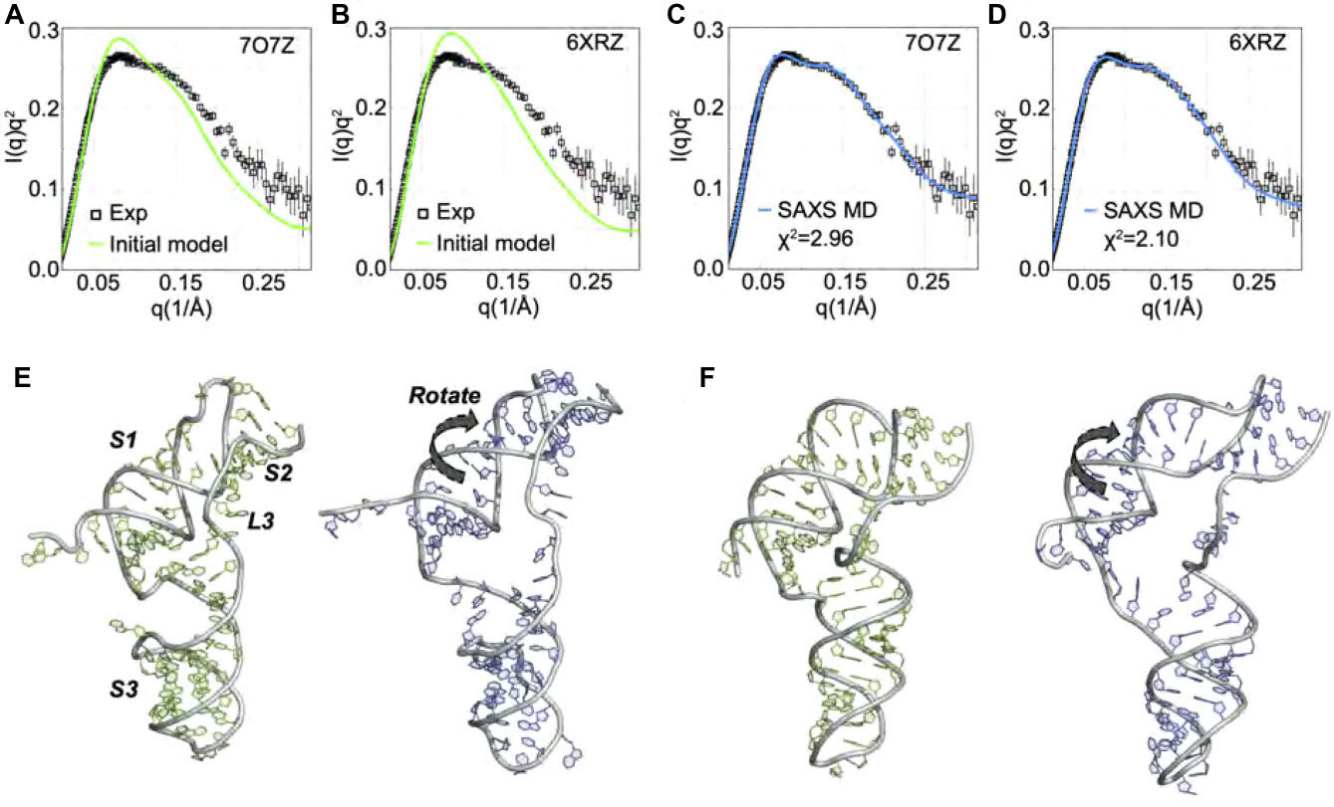
Comparison of A63C mutant of pseudoknot structure before and after SAXS-driven MD simulations. (**A**, **B**) Computed SAXS from the initial structures of PDBIDs 7O7Z and 6XRZ after introducing a single point mutation of A63C compared to the experimental profile. (**C**, **D**) Same comparison this time with the SAXS-driven MD pool. The computed χ^2^ measures the agreement. (**E**, **F**) A comparison of structures before (green) and after SAXS-Driven MD (blue) where the 7O7Z case is shown in panel (E) and 6XRZ is displayed in panel (F). The black arrows are guides to the eye to demonstrate the stem 1 reorientation after refinement.

To gain insight into the real-space structural changes that reflect these changes in the SAXS profile for the mutant, we performed SAXS-driven MD simulations comparable to those for the wild-type pseudoknot. To generate initial structures without experimental models, we replaced A63 in the PDB models of the wild-type pseudoknot with C. We limited the analysis to the two cryo-EM models, as they agreed best with the SAXS data for the wild-type. The SAXS profiles computed for these initial structures disagreed with the observed profile (Figure 8A, B), but after refinement by SAXS-driven MD the agreement was very good (Figure 8C, D); analysis of the residuals again indicated the consistency between the data and the fits (Supplementary Figure S10), and the resulting χ^2^ was even lower than for the wild-type pseudoknot.

**Figure 9.**
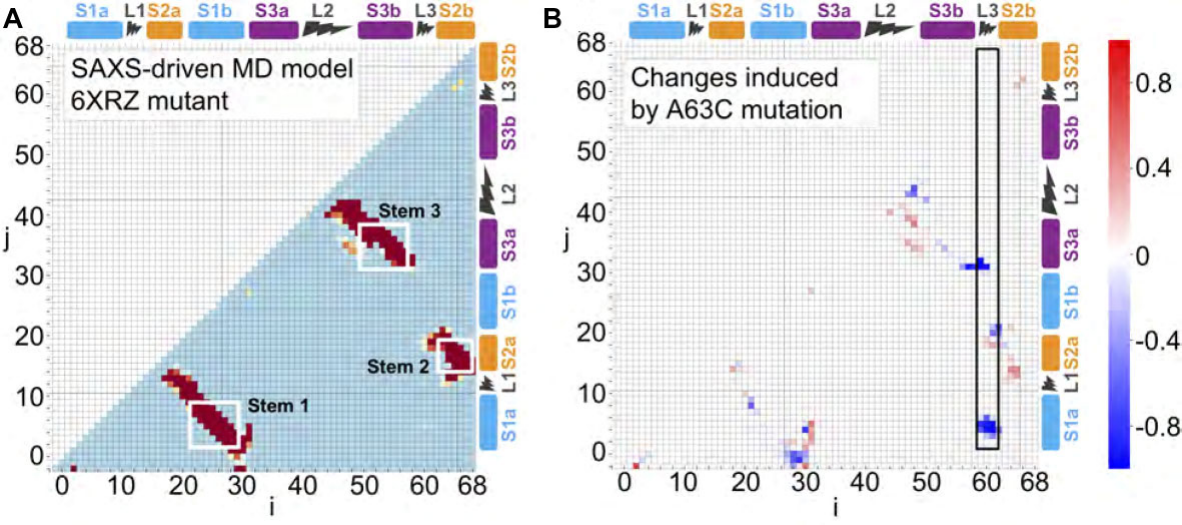
Contact maps for the 6XRZ constructs and the differences created by SAXS-driven MD and single point A63C mutation. (**A**) Similar to Figure 6A, the contact map of the SAXS-driven MD ensemble for mutant, where the regions of stem 1, stem 2 and stem 3 are highlighted and colors represent the different probability of contacts. (**B**) The differences created by A63C mutation when comparing the wild type to mutant, based on SAXS-driven MD.

Comparing the 3D structures for initial and final models (Figure 8E, F), we found that the refined structures featured a strong bend of S1 relative to S3, and an enlargement of the ring around the 3-helix junction owing to L3 moving away from the junction. These are the same trends seem in the modification to the wild-type structure. For the mutant, there was, in addition, a noticeable rotation of S1 relative to the junction. Analyzing the changes in more detail via the contact map for the refined structure starting from 6XRZ (Figure 9A), we found broadly similar features in the contact map as in the map for the corresponding refined wild-type pseudoknot structure (Figure 6A), but with some notable differences that could be seen clearly in the difference map between mutant and wild-type (Figure 9B). The changes were dominated by the loss of contacts involving S1 and L3, particularly in the latter. Contacts were significantly reduced between L3 and regions in S1a, S1b, and S3a. Contacts were also lost at both ends of S1, particularly near the 5′ end of the pseudoknot. In contrast, stronger contacts were observed between L3 and S2a, and within stem 2, suggesting that S2 becomes more compact and rigid. These changes also altered interactions in S3 and L2. Overall, these changes were consistent with a rotation of stem 1 modifying the relative positions of S1 and S2 as observed in the 3D images (Figure 8E, F).

## Discussion

By measuring the SAXS profile of the SARS-CoV-2 pseudoknot and comparing to the scattering profiles computed from published structures, this work explores how well the structural models obtained from cryo-EM and crystallography capture the conformation of an important frame-shifting pseudoknot under solution conditions. Intriguingly, none of the three published structures yielded solution scattering profiles consistent with the structural ensemble populated in the solution. The discrepancies were largest for the crystal structure but still notable for cryo-EM generated models, with the primary differences being a larger bend angle between S1 and S3 in the solution structures, and a more open ring around the 3-helix junction through which the 5′ end was threaded.

The discrepancies are most likely due to differences in measurement conditions used in the different assays and/or conformational selection effects in analysis. Whereas SAXS measurements were performed at monovalent ion concentrations near 130 mM, at pH 7.5, samples for cryo-EM of the isolated pseudoknot were prepared at high Mg^2 +^ (10 mM) and pH 8 (17), samples for cryo-EM of the pseudoknot on the ribosome were prepared at more modest Mg^2 +^ (5 mM) and pH 7.4 but with the ribosome stalled on the mRNA (19), and crystals were prepared from a mother liquor at acidic pH (5.5) with high Mg^2 +^ concentration (20 mM) that also contained 30–40% 2-methyl-2,4-pentanediol (MPD) and 1 mM cobalt hexamine, then transferred to a buffer containing 40% MPD, 30 mM Mg^2 +^, 1.5% DMSO, and 150 mM K^+^ (20). Crystal contacts may have played a role in favoring a linear-rather than bent-conformation, owing to more favorable packing, given that both of the cryo-EM structures do feature some bending of S3 with respect to S1. High Mg^2 +^ levels might favor a more compact ring by promoting interactions with L3, a feature seen in both cryo-EM and crystal structures. The presence of the ribosome must also account for some of the differences with 7O7Z, given that the ribosome makes specific contacts to the pseudoknot (19).

**Figure 10.**
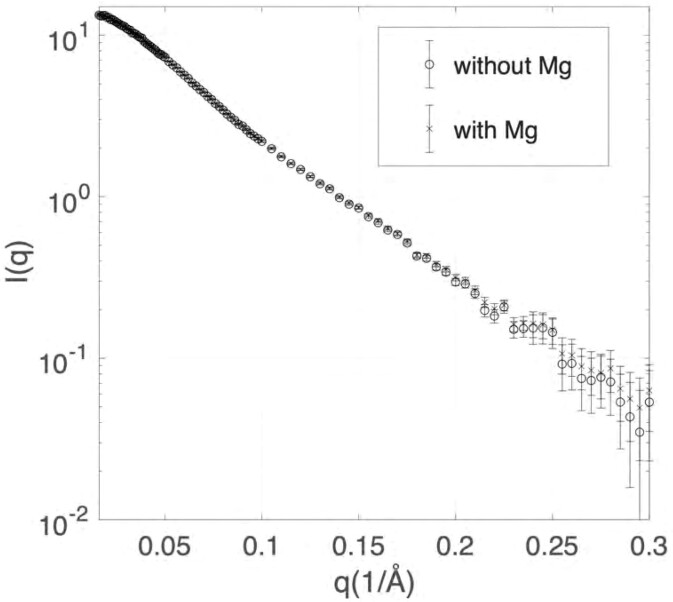
Comparison of wild type under conditions without and with Mg^2+^. The scattering profiles of wild type pseudoknot without and with 0.5 mM MgCl_2_.

The differences in Mg^2 +^ concentrations between the different assays are particularly notable. Because of the importance of Mg^2 +^ in RNA structures (45), we explored whether the SAXS profile might change when Mg^2 +^ was added to the buffer. With 0.5 mM Mg^2+^, near the lower end of the physiological range (46), we found that the SAXS profile was identical within noise to that obtained without Mg^2 +^ (Figure 10), implying that the pseudoknot conformation is not substantially changed at low levels of Mg^2+^. However, we were unable to test if higher concentrations changed the conformation, owing to Mg-induced dimerization at the high RNA concentrations needed for SAXS measurements, which altered the SAXS profile at low q (Supplementary Figure S12). Attempts to use SEC-SAXS with Mg-containing buffers were unsuccessful owing to excessive aggregation, and measurements using a proposed dimerization-resistant variant in which a dimerization domain in L2 is mutated (44) still showed signs of dimerization, albeit at reduced levels (Supplementary Figure S13).

Another key difference between the methods is that, unlike crystallography (where crystal symmetry suppresses heterogeneity) and cryo-EM (where only subsets of similar images are selected for analysis), SAXS measurements average over all the conformations sampled by the pseudoknot, without any selection of particular conformations.

The results for the non-frameshifting mutant show that the point mutation of A63 to C produces changes in conformation that are surprisingly large. Changes in the scattering are noticeable even in the raw profile. This mutation alters the base-pairing in S2 and forces a rotation of S1, which in turn enhances the bend between stems 1 and 3, while favoring a more open loop in L3. These effects suggest potential mechanisms for the strong influence of A63C on frameshifting levels. One possibility is that the rotation of S1 induced by A63C alters interactions with the ribosome that are important to frameshifting. For example, G12 in L1 makes a specific contact with ribosomal protein uS3 that is likely broken in the mutant owing to S1 rotation, and previous work showed that mutating G12 decreased frameshifting by up to 85% (19). Opening the ring around the 5′-threaded end might also contribute to a decrease in frameshifting by altering the effective length of the spacer between the pseudoknot and slippery sequence, to which frameshifting levels are sensitive (47), through increased flexibility of the ring constraining the 5′ end.

In conclusion, we used small angle X-ray scattering coupled with molecular dynamics simulations to elucidate the solution structure of the SARS-CoV-2 pseudoknot, an important conserved element of the viral genome that is a potential therapeutic target. Comparing the solution structure to the structures derived from cryo-EM and X-ray crystallography, we found distinct differences, as it was more bent and had a more open central ring. We also found the solution structure of the frameshift-inhibiting mutant A63C, observing major changes in the long-range contacts leading to the rotation of stem 1. These results help clarify inconsistencies between existing models of the SARS-CoV-2 pseudoknot, refine our understanding of its structure, and provide insight into how modifications to the pseudoknot may lead to changes in frameshifting.

## Supplementary Material

gkad809_Supplemental_Files

## Data Availability

Data associated with the reported analyses are available upon reasonable request from the corresponding author. All data necessary to assess the conclusions presented in this study are included in either the paper or the Supplementary Information. Additionally, the SAXS data has been deposited in the Small Angle Scattering Biological Data Bank (SASBDB) (https://www.sasbdb.org/) with the accession codes SASDRT6 (wildtype) and SASDRU6 (variant).
