# Supplementary material for: Atomistic structure of the SARS-CoV-2 pseudoknot in solution from SAXS-driven molecular dynamics
Source: Nucleic Acids Res. 2023 Oct 11;51(20):11332–44. doi: 10.1093/nar/gkad809 (PMC10639041; doi:10.1093/nar/gkad809)
Supplement: gkad809_Supplemental_Files [file gkad809_supplemental_files.zip › supporting_information (1).pdf]

Supplementary Information
Atomistic structure of the SARS-CoV-2 pseudoknot in solution from SAXS-driven molecular dynamics

Weiwei He,[†,‡,¶] Josue San Emeterio,[§,¶] Michael Woodside,[‖] Serdal Kirmizialtin,[*,†,‡] and Lois Pollack[*,§]

†*Chemistry Program, Science Division, New York University Abu Dhabi*

‡*Department of Chemistry, New York University*

¶*Equal contribution*

§*School of Applied and Engineering Physics, Cornell University*

‖*Department of Physics, University of Alberta*

E-mail: serdal@nyu.edu; lp26@cornell.edu

General MD simulations set up

The initial models of the wide-type SARS-CoV-2 pseudoknot, with a sequence of UUU GCG GUG UAA GUG CAG CCC GUC UUA CAC CGU GCG GCA CAG GCA CUA GUA CUG AUG UCG UAU ACA GGG CU, was built from the Cryo-EM models (PDB ID: 6XRZ[1]) and (PDB ID: 7O7Z[2]). As explained in the main text, the 5′ end of the crystallographic construct (PDB ID: 7LYJ[3]) was extended resulting in a sequence of UUU GCG GUG UAA GUG CAG CCC GUC UUA CAC CGU GCG GCA CAG GCC UAG UAC UGA UGU CGU AUA CAG GGC U. We did not include in our analysis the crystal structure obtained by co-crystallization with an antibody (PDB ID: 7MXL[4]), because the presence of the antibody and extensive mutations in L2 to generate the antibody-binding site made interpretation of SAXS-driven MD difficult. As the structure of the A63C mutant has not been solved

experimentally, we mutated the A63 to C computationally in both cryo-EM models ((PDB ID: 6XRZ, 7O7Z) to obtain the initial coordinates of mutant constructs (Fig. 1).

Table S1: Sequences of RNAs used in SAXS experiments

Transcribed sequence wild type	TAA TAC GAC TCA CTA TAG<br>GG TTT GCG GTG TAA GTG CAG CCC GTC TTA CAC CGT GCG<br>GCA CAG GCA CTA GTA CTG ATG TCG TAT ACA GGG CTT TTT
Transcribed sequence mutant	TAA TAC GAC TCA CTA TAG<br>GG TTT GCG GTG TAA GTG CAG CCC GTC TTA CAC CGT GCG<br>GCA CAG GCA CTA GTA CTG ATG TCG TAT ACC GGG CTT TTT
Resulting sequence wild type	GGG UUU GCG GUG UAA GUG CAG CCC GUC UUA CAC CGU GCG<br>GCA CAG GCA CUA GUA CUG AUG UCG UAU ACA GGG CUU UUU
Resulting sequence mutant	GGG UUU GCG GUG UAA GUG CAG CCC GUC UUA CAC CGU GCG<br>GCA CAG GCA CUA GUA CUG AUG UCG UAU ACC GGG CUU UUU

Table S2: Sequences of RNAs used in SAXS-driven MD

6XRZ wild type	UUU GCG GUG UAA GUG CAG CCC GUC UUA CAC CGU GCG<br>GCA CAG GCA CUA GUA CUG AUG UCG UAU ACA GGG CU
6XRZ mutant	UUU GCG GUG UAA GUG CAG CCC GUC UUA CAC CGU GCG<br>GCA CAG GCA CUA GUA CUG AUG UCG UAU ACC GGG CU
7O7Z wild type	UUU GCG GUG UAA GUG CAG CCC GUC UUA CAC CGU GCG<br>GCA CAG GCA CUA GUA CUG AUG UCG UAU ACA GGG CU
7O7Z mutant	UUU GCG GUG UAA GUG CAG CCC GUC UUA CAC CGU GCG<br>GCA CAG GCA CUA GUA CUG AUG UCG UAU ACC GGG CU
7LYJ wild type	UUU GCG GUG UAA GUG CAG CCC GUC UUA CAC CGU GCG<br>GCA CAG GC CUA GUA CUG AUG UCG UAU ACA GGG CU

The structure of pseudoknot was placed in a simulation box such that the shortest distance from the RNA surface to the edges is at least 20Å resulting a simulation box of 10.0x10.0x14.0 nm^3. We added water and ions to mimic the salt conditions of the experiments. We used TIP3P[5] to represent water. RNA is represented by χOL3 forcefield.[6] For ions we used the smith and dang parameters.[7] MD simulations were carried out using GROMACS 5.0.5 suite[8]. In addition to the RNA simulations we prepared buffer boxes comprise explicit water and ions matching the bulk salt conditions used in our experiments. Table S3 below summarizes all the simulation set ups and with the number of solvent molecules used.

To accurately compute the intermolecular interactions, the long-range electrostatic interactions were treated using the particle mesh Ewald[9] (PME) summation method with a grid spacing of 0.12 nm and an interpolation of order 4. We used periodic boundary conditions in all directions. For van der Waals forces we used a dispersion correction[10]. A distance cutoff of 1.1 nm was considered for electrostatic and van der Waals interactions with the

same cutoff for neighbor search. We updated the neighbor lists every 40 steps. The covalent bonds of the water molecules and RNA were constrained by SETTLE[11] and LINCS[12] algorithms, respectively. The equations of motion were solved by leap-frog integrator[13] using an integration time step of 2 fs.

Table S3: Simulation setup details of RNA systems understudy

	6XRZ wild type	6XRZ mutant	7O7Z wild type	7O7Z mutant	7LYJ wild type
Simulation box	207 K^+ 137 Cl^- 44737 H_2O	207 K^+ 137 Cl^- 44737 H_2O	207 K^+ 137 Cl^- 44735 H_2O	207 K^+ 137 Cl^- 44738 H_2O	207 Na^+ 138Cl^- 44752 H_2O
Buffer box	151 KCl 45579 H_2O	151 KCl 45579 H_2O	151 KCl 45579 H_2O	151 KCl 45579 H_2O	151 KCl 45579 H_2O

Simulation box: RNA+water+ion; Buffer box: water+ion. (see Fig. S1)

For each constructed system, we removed the bad contacts resulting from the random placement of water and ions by performing 5000-step steepest descent energy minimization. Subsequently, we employed a 5-ns-long simulation at an NPT ensemble, at a constant pressure of 1 bar and temperature of 300K, to determine the volume of the simulation box and relax water and ions around RNA. During this simulation, we restrained the RNA molecules using harmonic restraints implemented in Gromacs with force constants of ($1000kJ \cdot mol^{-1} \cdot nm^{-2}$) while water and ions were allowed to move. Later, we performed another 100 ns long constrained MD at canonical ensemble (NVT) to relax ions. Coordinates of the last snapshot were saved and used as the starting structures for subsequent 100-ns long unrestrained simulations where we generate the initial conformational pools. We select the conformation with lowest χ^2 error from the pool as best fit. The best fit structures were later used to start SAXS-driven MD simulations which will be explained below.

Computing SAXS profiles from MD trajectory

We calculated the theoretical SAXS profiles from the simulation following the previous studies,[14–16] this methodology was shown to align well with experimental data.[17–19] In short, we compute the contrast of electron density between the solute (RNA+ion+water system, Fig. S1) and the solvent (ion+water system, Fig. S1) separately. Then the buffer-subtracted scattering intensity is obtained by $I(q) = I_A(q)$-$I_B(q)$, where I_A and I_B denote the detected SAXS signals of the sample solution (RNA+ion+water system) and solvent background (ion+water system). We use a spatial envelope of d=10Å to enclose the ions shell and water near the pseudoknot surface. The solvent environment beyond this molecular isosurface is assumed to be bulklike. The scattering of the sample solution ($I_A(q)$) was estimated from this molecular envelope. To estimate the electron density of the solvent background ($I_B(q)$) and the excluded volumes, 20-ns-long MD simulations of ions+water system were performed in NVT ensemble. This buffer simulation contains the ion pairs and water molecules in a periodic box with dimensions and ionic strength matching the RNA+ion+water system. The same envelope was applied to bulk-solvent system for buffer subtractions.

SAXS-driven MD simulations

To set up the SAXS-driven MD, we used stochastic dynamics (SD) integrator.[17,18,20] During the conformational sampling, we removed the center of mass motion, while the other settings remained unchanged from the previous section of **General MD simulation set up**. Referring to our previous work,[17,18] the memory time τ was set to 250 ps which ensured the convergence of buffer subtraction. The time interval t was set to 15 ns for the switch function $\alpha(t)$ so that sufficient convergence of $I_{com}(q, \mathbf{R}, t)$ was guaranteed before adding the energetic penalty. The construct was refined against the SEC-SAXS data up to $q = 3.15\ nm^{-1}$ and we set n_q=50 in our study. 1500 q-vectors were used for computing orientational average of scattering intensity. The coupling constant k_c, is adjusted depending on the system and the extend of deviation between model and experimental measurement. Namely, we set it to 1.0 for both wild type and mutant of 6XRZ, 5.0 for the simulations of both 7O7Z and 7LYJ. The computed SAXS curves were reported every 5 ps to monitor the progress of conformational search. We conduct 200 ns long simulations. We analyze the convergence of simulation by monitoring χ^2 (Fig. S4). The conformations $\chi^2 < 4$ were collected and used to construct structural pools for data analysis.

Data analysis

Contact map analysis

To investigate the structures and differences between them, we employed contacts map analysis. For a given RNA conformation, at time t, the contacts is considered between residue i and j when the shortest distance between residues in time $s_{ij}(t) < r_c$, where r_c is the critical distance for contact formation and is set to 8Å . We exclude neighbors by setting $|i - j| > 3$. The average contact formation probability $C(i, j)$ then becomes

$$C(i, j) = \frac{1}{L} \int \Theta(r_c - s_{ij}(t))dt \qquad (1)$$

where $\Theta(x)$ is the Heaviside function and L is the simulation length set to 200 ns in our study.

Similarly, we compute the difference in contact map probabilities between two structural pool, defined as $\delta C_{1,2}(i, j) = C_1(i, j) - C_2(i, j)$, here $C_1(i, j), C_2(i, j)$ are contact probabilities of pair i and j from model 1 and 2 respectively.

Principal component analysis

To investigate the essential degrees of freedom modified during the SAXS-Driven MD refinement, we used the principal component analysis (PCA) implemented in Gromacs[21]. The calculation of the eigenvectors and eigenvalues, and the projection of the given trajectory along the first two principal components was performed using *covar* and *anaeig* of Gromacs utilities. For that, all atoms of the RNA construct were used in performing the PCA and the entire 200 ns long trajectory is used by monitoring the atomic fluctuations of each residue.

The eigenvectors of the two highest eigenvalues (PCA vec 1 and PCA vec 2) were projected and displayed in Fig. S5.

The effect of Mg^{2+} on SAXS profiles

To determine if the results depended on the presence of Mg^{2+} ions in the measuring buffer, SAXS profiles for the wild-type pseudoknot were measured at 3 different concentrations of $MgCl_2$ added to the buffer: 0.5 mM, 1.0 mM, and 4.0 mM (Fig. S12). Attempts to use SEC-SAXS to separate out the monomeric fraction were unsuccessful, as there was too much aggregation when using SEC columns with buffers containing $MgCl_2$. Measurements of a variant with the dimerization domain mutated away (residues AGU in L2 mutated to UCC) still showed dimerization at higher $MgCl_2$ concentrations (Fig. S13), suggesting some of the dimzerization is non-specific.

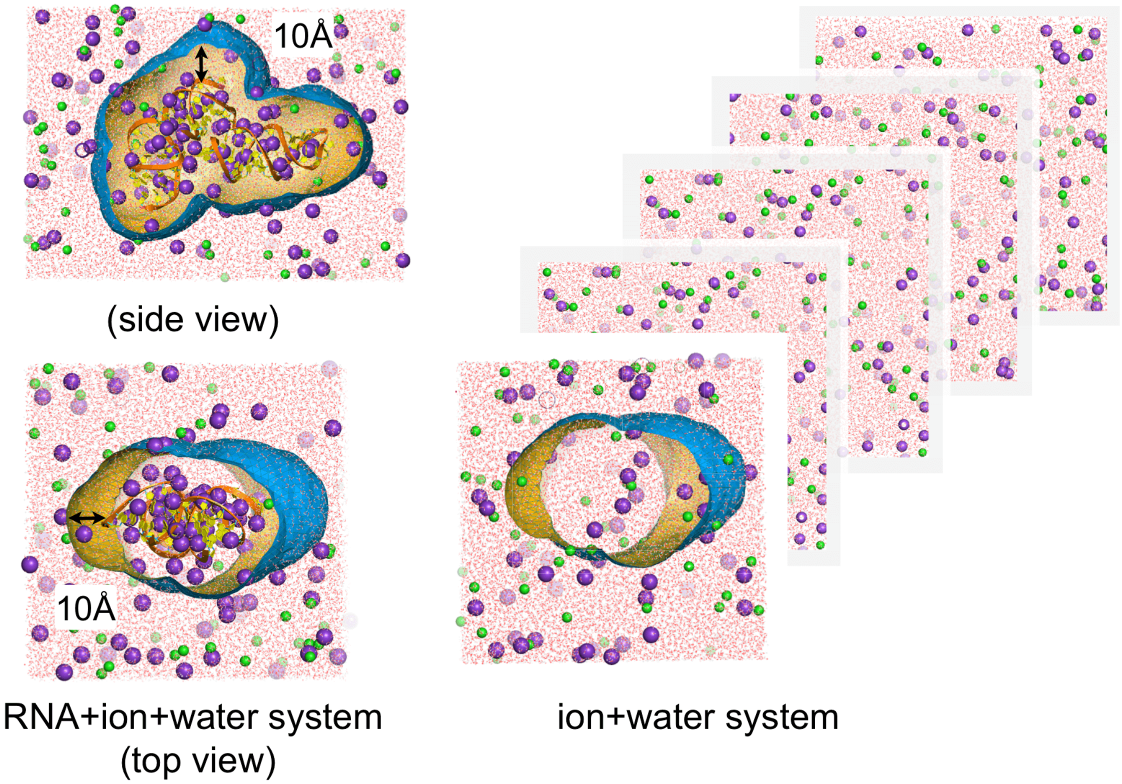

Figure S1: **Illustration of the RNA+ion+water and water+ion systems used to compute SAXS.** The molecular envelope, with 10 $\AA$ separation to the pseudoknot's surface, is constructed to encompass both ions and solvent. The scattering intensity of the water+ion system was computed by applying the same envelope. We subtract the scattering of the RNA-ion system from solvent as described in Section **Computing SAXS from MD trajectory**.

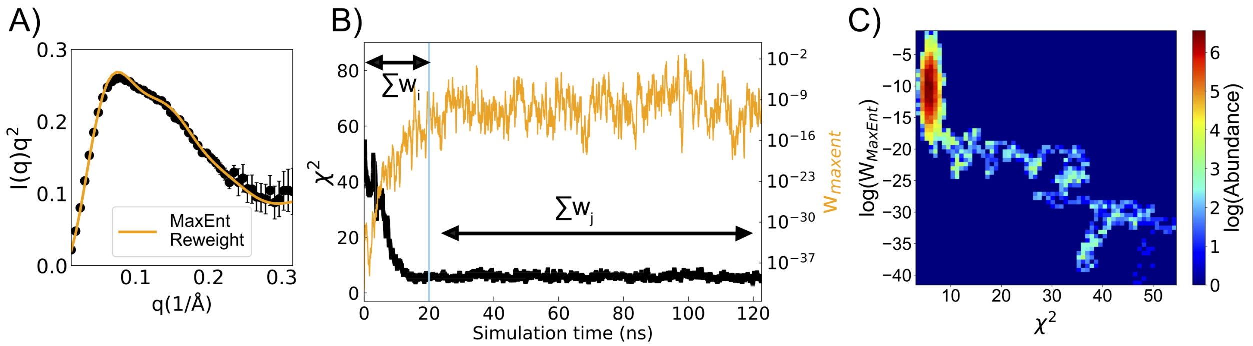

Figure S2: **Maximum entropy ensemble refinement of the SAXS-driven conformation pool.** (A) Fitting of SAXS profile maximum entropy ensemble refinement re-weighting. (B) The weights of conformations in the regions of the simulation with $\chi^2 > 4$ (left of blue line) and $\chi^2 < 4$ (right of blue line). (C) Heatmap of the weights of the conformations as a function of χ^2, the colorbar indicates the relative abundance, with blue color representing low and red color representing high abundance.

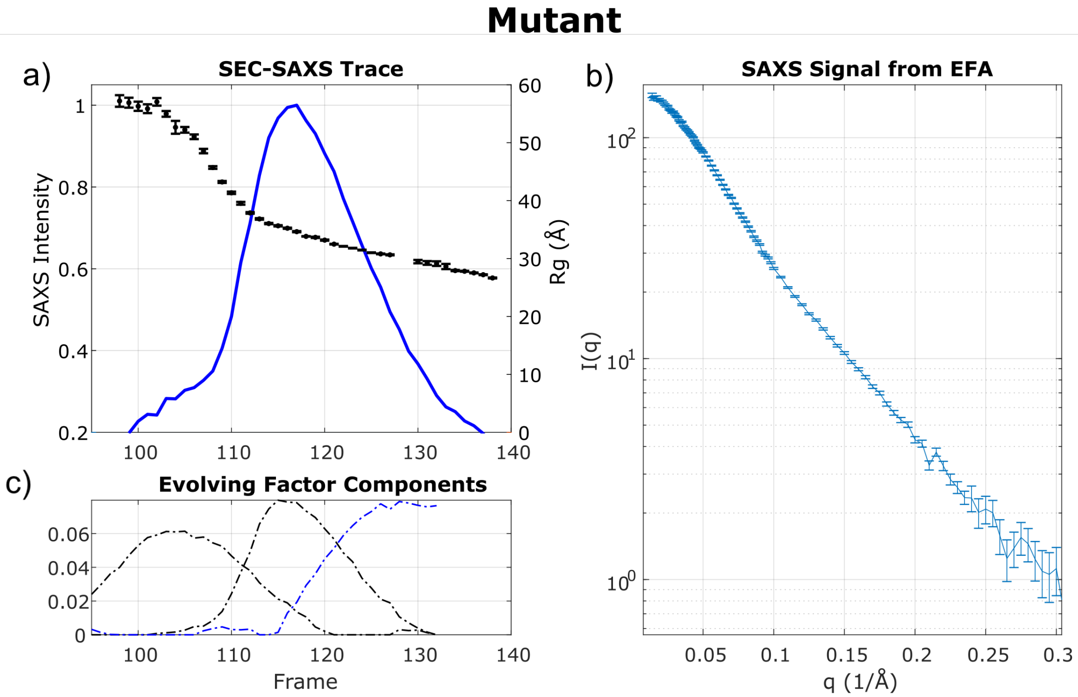

Figure S3: **Size exclusion chromatography SAXS data of Mutant construct.** a) Shown is the size exclusion trace from the integrated SAXS intensity (left axis) overlayed with the radius of gyration (right axis). The components extracted through evolving factor analysis are shown below c). The peaks corresponding to the monomer fractions are shown in blue. The extracted SAXS profile is shown to the right b).

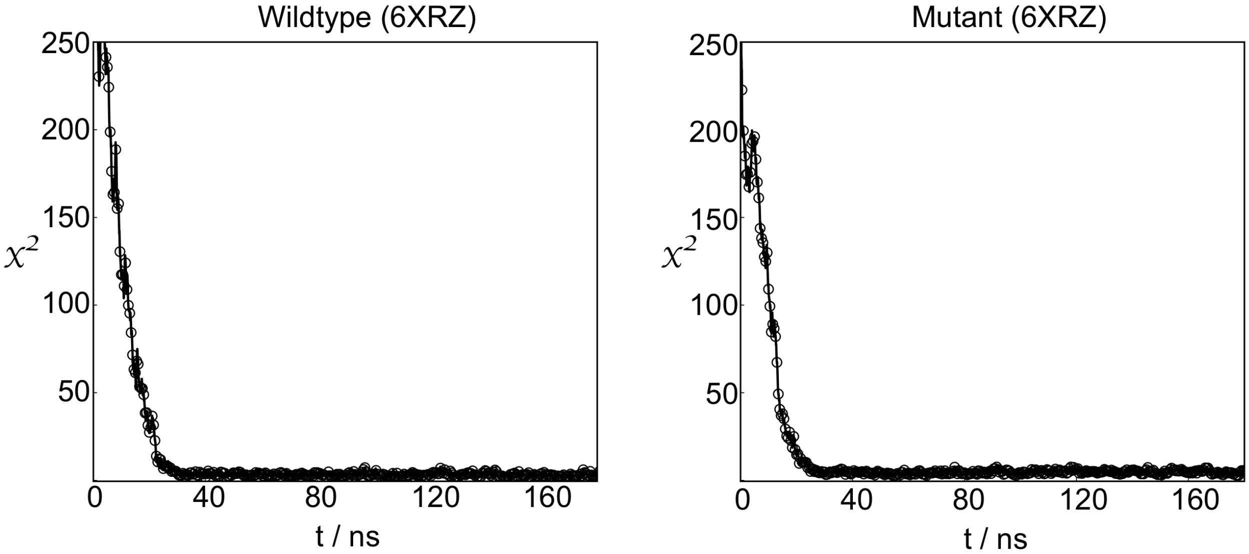

Figure S4: **Time evolution of χ^2 during SAXS-driven MD simulations.** Wild type (left) and mutant (right) of 6XRZ model are shown. Here, the asymptotic value of the χ^2 curve indicates the convergence of conformational searching at t$\sim$ 20ns.

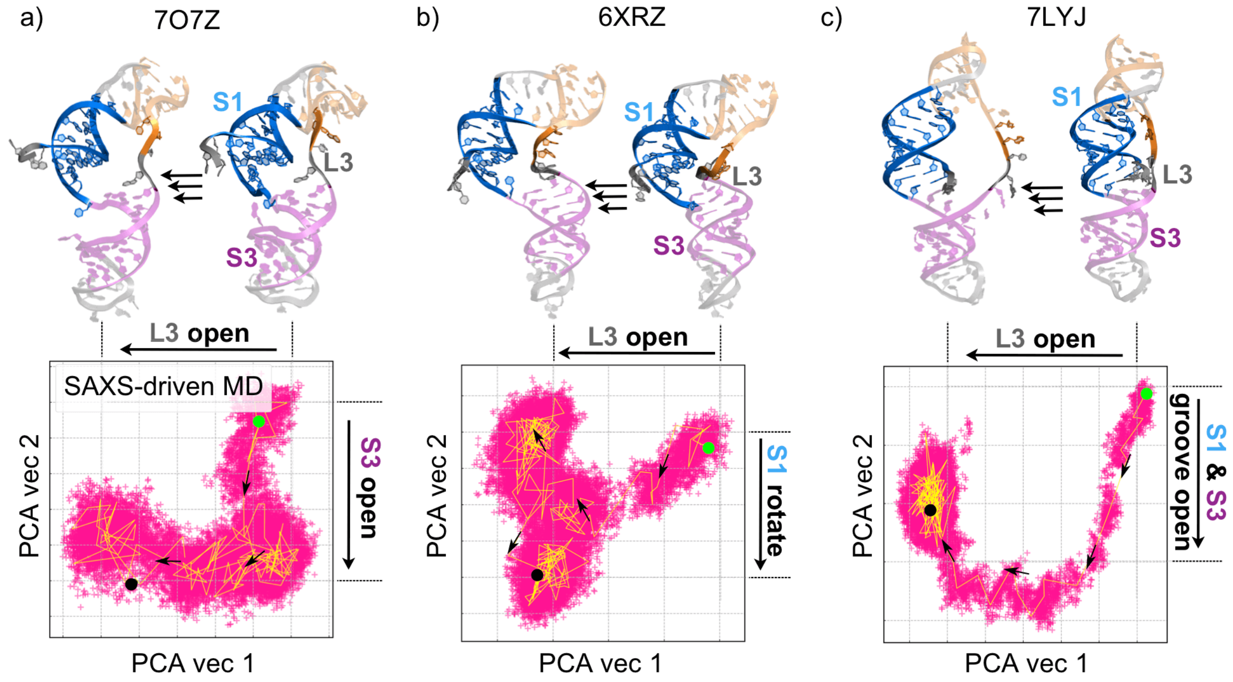

Figure S5: **Principle component analysis of SAXS-driven MD simulations.** PCA vector 1 approximately corresponds to the same motions of L3 open, represented by the insets on the top, in all cases a) 7O7Z, b) 6XRZ and c) 7LYJ. The conformational changes along PCA vector 2 vary with models, but mainly located on S1 or S3.

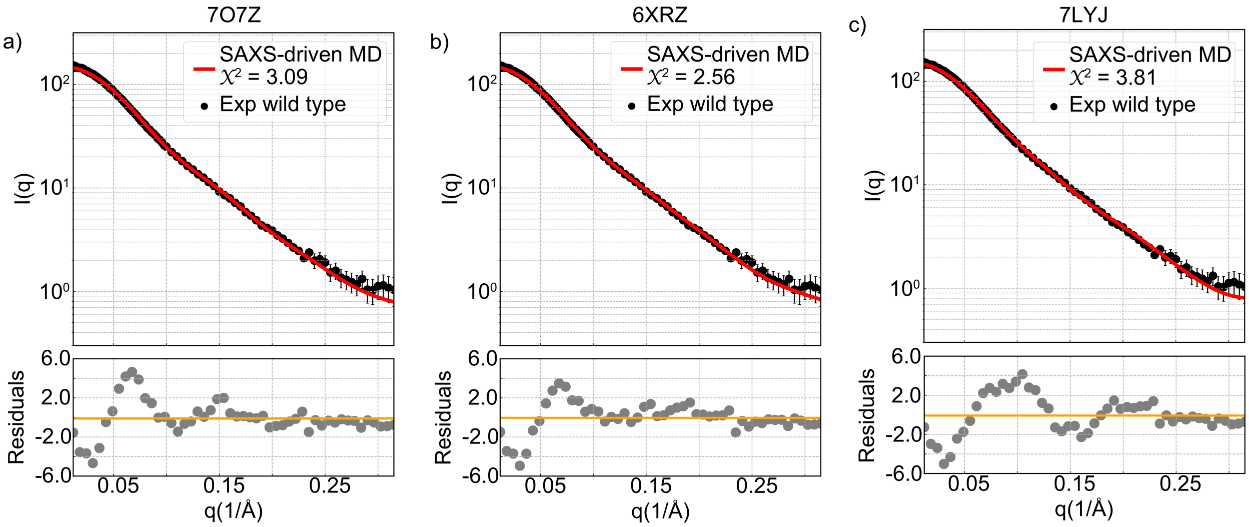

d)

Statistics	7O7Z	6XRZ	7LYJ
Z	5.427	5.830	6.634
p-value	5.715e-8	5.560e-9	3.099e-11

Figure S6: **Residuals between the calculated SAXS curves and the experimental SAXS curves, evaluated at the q-points applied during the SAXS-driven simulations of 7O7Z, 6XRZ and 7LYJ** a) 7O7Z. b) 6XRZ. c) 7LYJ. Wald-Wolfowitz runs test is shown in d).

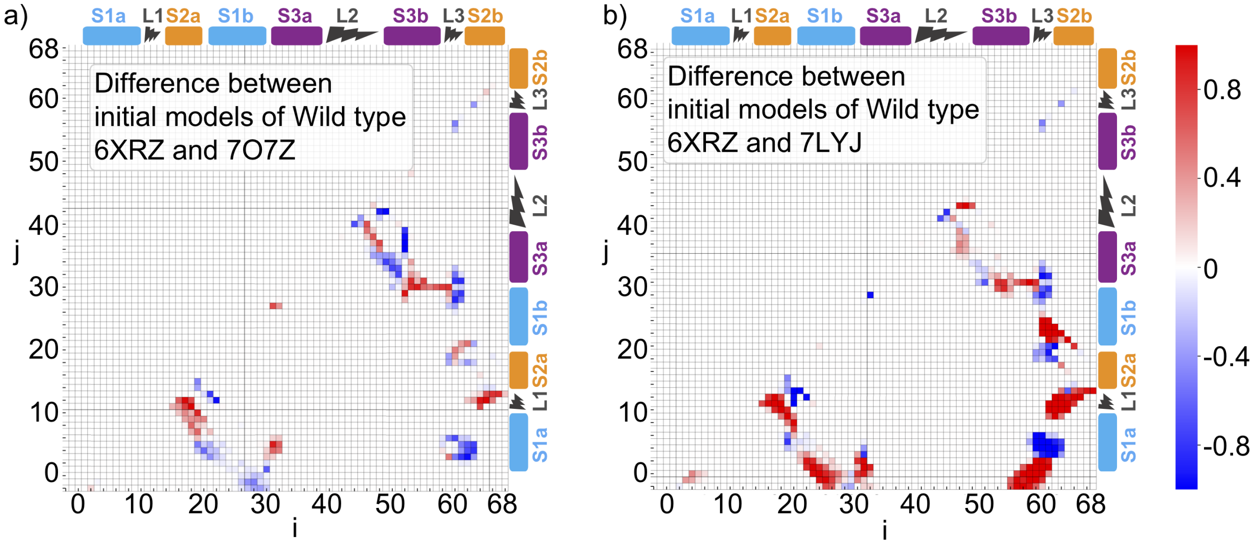

Figure S7: **The difference map of comparing initial models from cryoEM and crystallography.** a) The structural difference between RNA-only cryoEM 6XRZ and Ribosome-bound cryoEM 7O7Z. b) The structural difference between RNA-only cryoEM 6XRZ and crystal 7LYJ.

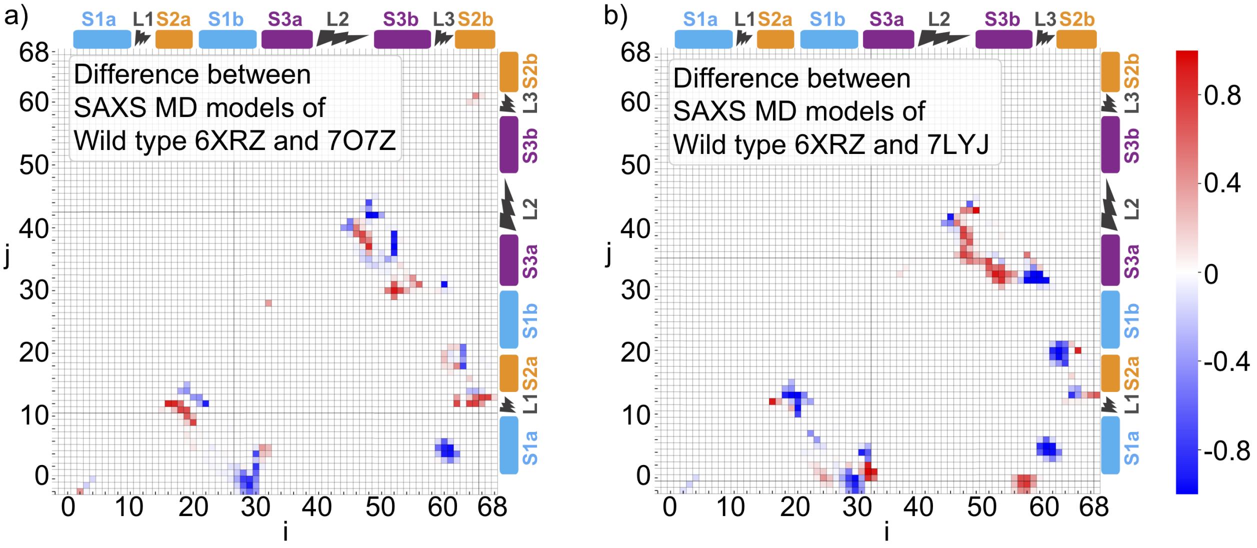

Figure S8: **The difference map of comparing solution structures derived from different experiments by SAXS-driven MD.** a) The structural difference between solution structure derived from RNA-only cryoEM 6XRZ and the one derived from Ribosome-bound cryoEM 7O7Z. b) The structural difference between solution structure derived from RNA-only cryoEM 6XRZ and the one derived from crystal 7LYJ. In contrast to Fig. S7, after refinement by SAXS-driven MD, the conformational disparity reduced significantly.

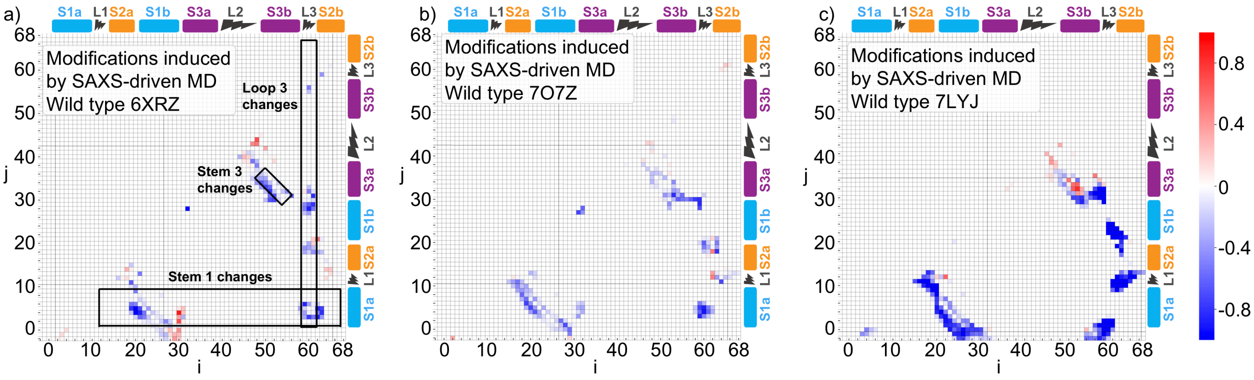

Figure S9: **The difference map of comparing experimentally resolved structures and SAXS-driven MD derived solution structures.** a) The structural difference between RNA-only cryoEM structure, 6XRZ and the solution structure derived from it. b) The structural difference between Ribosome-bound cryoEM structure, 7O7Z and the solution structure derived from it. c) The structural difference between crystal structure, 7LYJ and the solution structure derived from it.

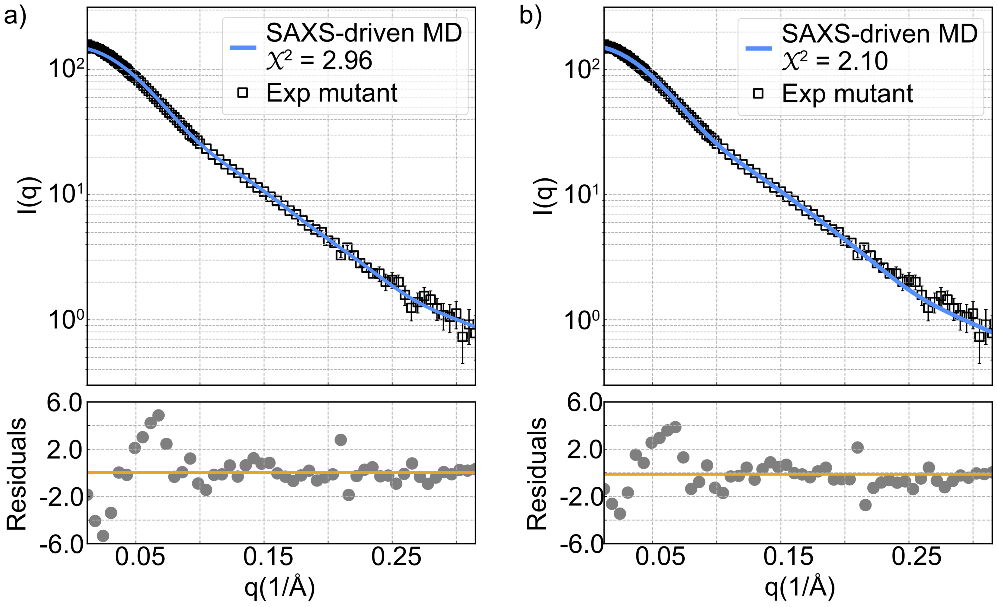

c)

Statistics	7O7Z	6XRZ
Z	4.422	6.031
p-value	9.761e-6	1.634e-9

Figure S10: **Residuals between the calculated SAXS curves and the experimental SAXS curves, evaluated at the q-points applied during the SAXS-driven simulations of 7O7Z and 6XRZ** a) 7O7Z. b) 6XRZ. c) Wald-Wolfowitz runs test.

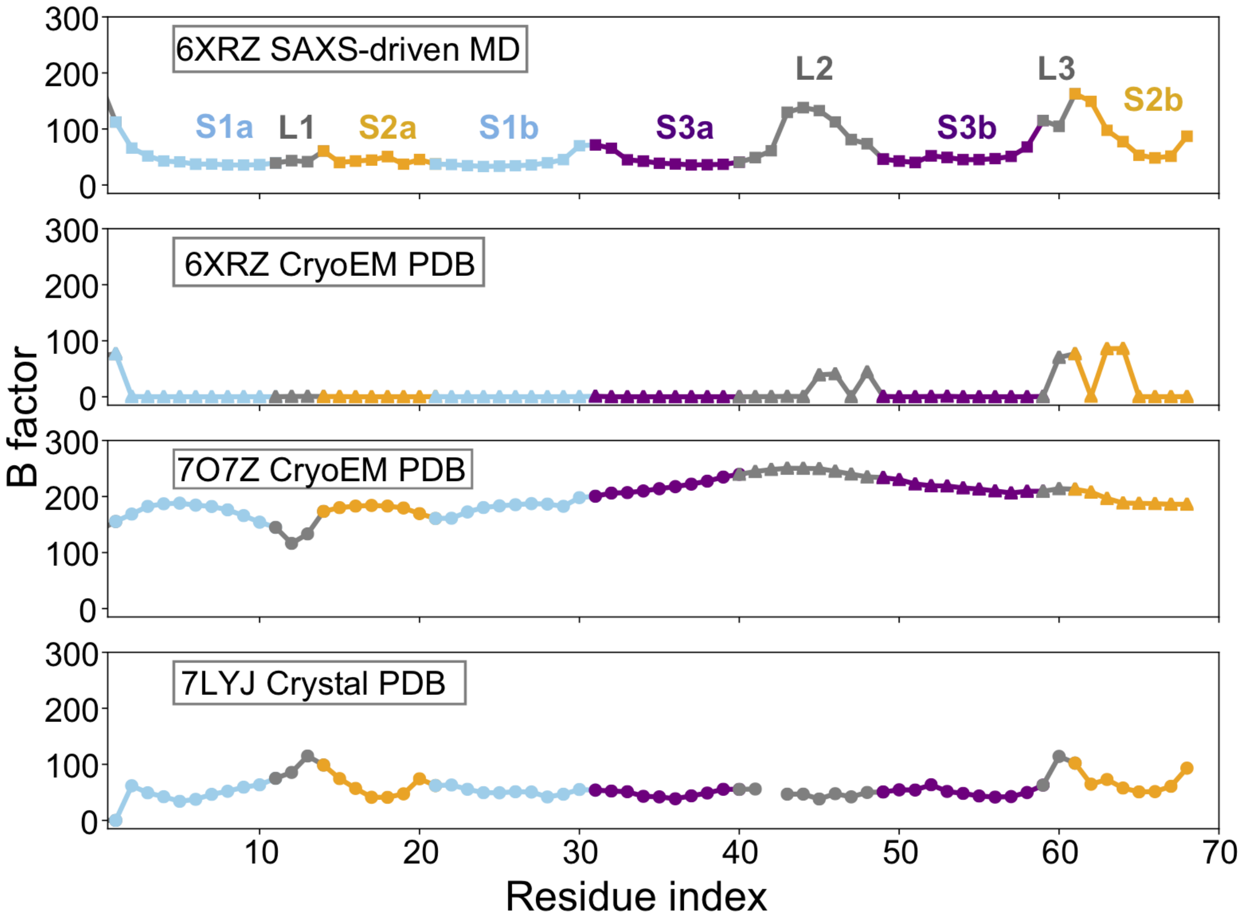

Figure S11: **B-factor as a function of residue index.** Flexibility of structures as reflected by B-factors for SAXS-driven MD model from 6XRZ (top panel), original 6XRZ structure (2nd panel), 7O7Z structure (3rd panel), and 7LYJ structure (bottom panel).

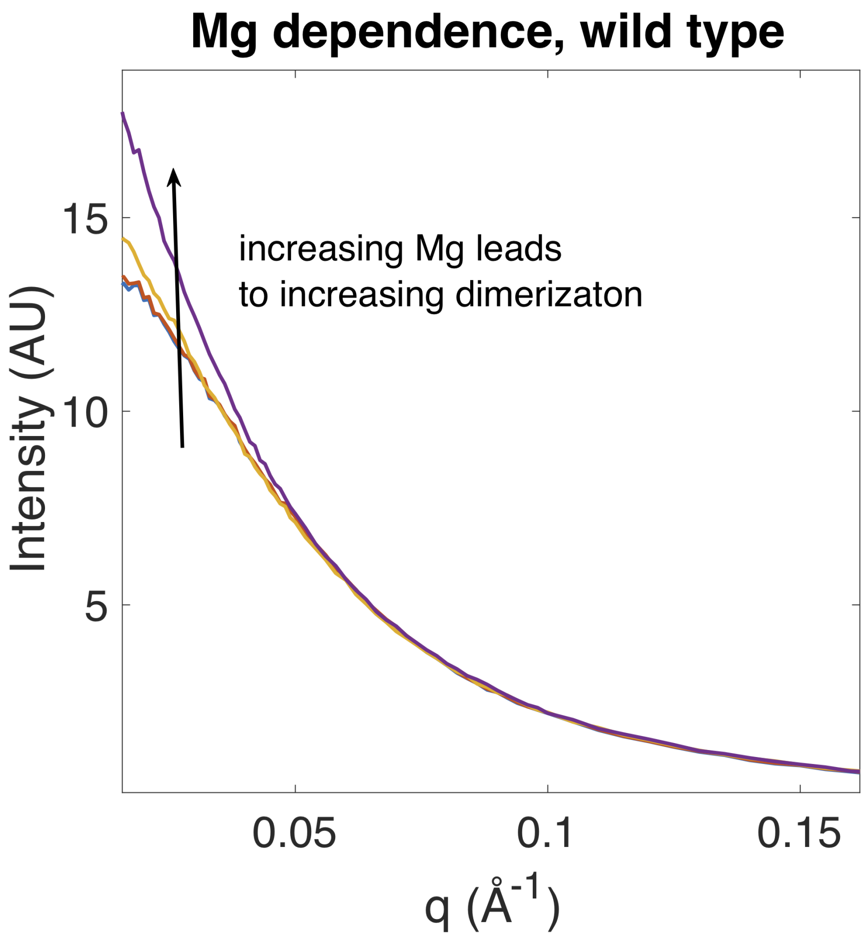

Figure S12: **SAXS profiles at varying added $MgCl_2$ concentrations.** SAXS profiles, focusing on the low-q region, show increased scattering at low q owing to molecular association (dimerization) as the $MgCl_2$ concentration increases (blue: 0 mM, red: 0.5 mM, yellow: 1 mM, purple: 4 mM).

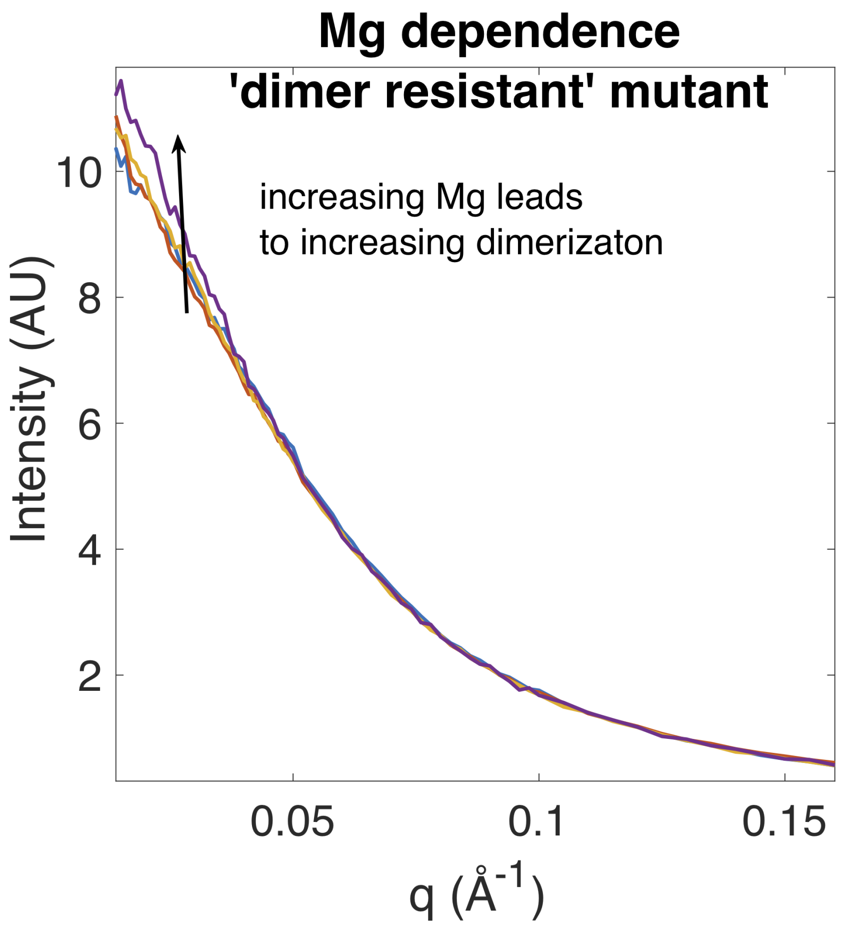

Figure S13: **SAXS profiles for dimerization-resistant mutant.** Mutating the dimerization domain in L2 reduces but does not eliminate dimerization at higher added $MgCl_2$ concentrations (blue: 0 mM, red: 0.5 mM, yellow: 1 mM, purple: 4 mM).

References

(1) Zhang, K.; Zheludev, I. N.; Hagey, R. J.; Haslecker, R.; Hou, Y. J.; Kretsch, R.; Pintilie, G. D.; Rangan, R.; Kladwang, W.; Li, S., et al. Cryo-EM and antisense targeting of the 28-kDa frameshift stimulation element from the SARS-CoV-2 RNA genome. *Nature Structural & Molecular Biology* **2021**, 1–8.

(2) Bhatt, P. R.; Scaiola, A.; Loughran, G.; Leibundgut, M.; Kratzel, A.; Meurs, R.; Dreos, R.; O'Connor, K. M.; McMillan, A.; Bode, J. W., et al. Structural basis of ribosomal frameshifting during translation of the SARS-CoV-2 RNA genome. *Science* **2021**, *372*, 1306–1313.

(3) Jones, C. P.; Ferré-D'Amaré, A. R. Crystal structure of the severe acute respiratory

syndrome coronavirus 2 (SARS-CoV-2) frameshifting pseudoknot. *RNA* **2022**, *28*, 239–249.

(4) Roman, C.; Lewicka, A.; Koirala, D.; Li, N.-S.; Piccirilli, J. A. The SARS-CoV-2 programmed- 1 ribosomal frameshifting element crystal structure solved to 2.09 Å using chaperone-assisted RNA crystallography. *ACS Chemical Biology* **2021**, *16*, 1469–1481.

(5) Jorgensen, W. L.; Chandrasekhar, J.; Madura, J. D.; Impey, R. W.; Klein, M. L. Comparison of simple potential functions for simulating liquid water. *The Journal of chemical physics* **1983**, *79*, 926–935.

(6) Zgarbová, M.; Otyepka, M.; Šponer, J.; Mládek, A.; Banáš, P.; Cheatham, T. E.; Jurečka, P. Refinement of the Cornell et al. nucleic acids force field based on reference quantum chemical calculations of glycosidic torsion profiles. *Journal of chemical theory and computation* **2011**, *7*, 2886–2902.

(7) Smith, D. E.; Dang, L. X. Computer simulations of NaCl association in polarizable water. *The Journal of Chemical Physics* **1994**, *100*, 3757–3766.

(8) Hess, B.; Kutzner, C.; Van Der Spoel, D.; Lindahl, E. GROMACS 4: algorithms for highly efficient, load-balanced, and scalable molecular simulation. *Journal of chemical theory and computation* **2008**, *4*, 435–447.

(9) Darden, T.; York, D.; Pedersen, L. Particle mesh Ewald: An N log (N) method for Ewald sums in large systems. *The Journal of chemical physics* **1993**, *98*, 10089–10092.

(10) Shirts, M. R.; Mobley, D. L.; Chodera, J. D.; Pande, V. S. Accurate and efficient corrections for missing dispersion interactions in molecular simulations. *The journal of physical chemistry B* **2007**, *111*, 13052–13063.

(11) Miyamoto, S.; Kollman, P. A. Settle: An analytical version of the SHAKE and RATTLE algorithm for rigid water models. *Journal of computational chemistry* **1992**, *13*, 952–962.

(12) Hess, B.; Bekker, H.; Berendsen, H. J.; Fraaije, J. G. LINCS: a linear constraint solver for molecular simulations. *Journal of computational chemistry* **1997**, *18*, 1463–1472.

(13) Hockney, R. W.; Goel, S.; Eastwood, J. Quiet high-resolution computer models of a plasma. *Journal of Computational Physics* **1974**, *14*, 148–158.

(14) He, W.; Chen, Y.-L.; Pollack, L.; Kirmizialtin, S. The structural plasticity of nucleic acid duplexes revealed by WAXS and MD. *Science Advances* **2021**, *7*, eabf6106.

(15) Park, S.; Bardhan, J. P.; Roux, B.; Makowski, L. Simulated x-ray scattering of protein solutions using explicit-solvent models. *The Journal of chemical physics* **2009**, *130*, 04B607.

(16) Chen, P.-c.; Hub, J. S. Validating solution ensembles from molecular dynamics simulation by wide-angle X-ray scattering data. *Biophysical journal* **2014**, *107*, 435–447.

(17) He, W.; Henning-Knechtel, A.; Kirmizialtin, S. Visualizing RNA Structures by SAXS-Driven MD Simulations. *Front. Bioinform. 2: 781949. doi: 10.3389/fbinf* **2022**,

(18) Chen, Y.-L.; He, W.; Kirmizialtin, S.; Pollack, L. Insights into the structural stability of major groove RNA triplexes by WAXS-guided MD simulations. *Cell Reports Physical Science* **2022**, *3*, 100971.

(19) He, W.; Naleem, N.; Kleiman, D.; Kirmizialtin, S. Refining the RNA Force Field with Small-Angle X-ray Scattering of Helix–Junction–Helix RNA. *The journal of physical chemistry letters* **2022**, *13*, 3400–3408.

(20) Chen, P.-c.; Hub, J. S. Interpretation of solution x-ray scattering by explicit-solvent molecular dynamics. *Biophysical journal* **2015**, *108*, 2573–2584.

(21) Amadei, A.; Linssen, A. B.; Berendsen, H. J. Essential dynamics of proteins. *Proteins: Structure, Function, and Bioinformatics* **1993**, *17*, 412–425.
